# *Tremella fuciformis* Polysaccharides Inhibited Colonic Inflammation in Dextran Sulfate Sodium-Treated Mice *via* Foxp3+ T Cells, Gut Microbiota, and Bacterial Metabolites

**DOI:** 10.3389/fimmu.2021.648162

**Published:** 2021-04-01

**Authors:** Yingyin Xu, Liyuan Xie, Zhiyuan Zhang, Weiwei Zhang, Jie Tang, Xiaolan He, Jie Zhou, Weihong Peng

**Affiliations:** ^1^ National-Local Joint Engineering Laboratory of Breeding and Cultivation of Edible and Medicinal Fungi, Soil and Fertilizer Institute, Sichuan Academy of Agricultural Sciences, Chengdu, China; ^2^ Scientific Observing and Experimental Station of Agro-microbial Resource and Utilization in Southwest China, Ministry of Agriculture, Chengdu, China; ^3^ Institute of Medicinal Plant Development, Chinese Academy of Medical Sciences & Peking Union Medical College, Beijing, China

**Keywords:** colitis, gut microbiota, inflammatory responses, microbial metabolites, *Tremella fuciformis* polysaccharides

## Abstract

*Tremella fuciformis* is an edible medicinal mushroom, and its polysaccharide components are found to confer various health benefits. This study identified the protective effects of polysaccharides of *Tremella fuciformis* (TPs) against dextran sulfate sodium (DSS)-induced colitis in mice. High dose of TPs (HTPs) could prevent the colon from shortening, reduce activity of colonic myeloperoxidase and serum diamine oxidase (DAO), decrease the concentration of D-lactate, and alleviate the colonic tissue damage in colitic mice. HTPs treatment stimulated Foxp3+T cells, and promoted the production of anti-inflammatory cytokines whereas it reduced the production of pro-inflammatory and the portion of immunoglobulin A (IgA)-coated bacteria, which was related to modulation of immune responses. 16S rRNA sequencing analysis showed that TPs could significantly increase gut community diversity, and restore the relative abundances of Lactobacillus, Odoribacter, Helicobacter, Ruminococcaceae, and Marinifilaceae. According to metabolomic analysis, HTPs induced specific microbial metabolites akin to that in normal mice. Tyrosine biosynthesis, tryptophan metabolism, and bile acid metabolism were influenced in the HTPs group compared with those in the DSS group. HTPs could alleviate DSS-induced colitis by immunoregulation and restored the gut microbiota and microbial metabolites. The results indicated that HTPs have potential to be developed as a food supplement to ameliorate intestinal diseases.

## Introduction

Inflammatory bowel disease (IBD) including ulcerative colitis and Crohn’s disease (CD) is a lifelong illness which poses serious threats such as chronic malabsorption of nutrients, abnormal pain, and rectal bleeding to human health. Even though the pathogenesis of IBD remains unclear, it is accepted that the interruption of the host immunity-microbiota interaction plays an essential role in the process of IBD development (1, 2). Regulatory T cells (Tregs) are critical in maintaining immune tolerance and suppressing autoimmunity, and transcription factor Foxp3 serves as a master switch for Treg (3). Immunoglobulin A (IgA) is the major effector molecule of the adaptive immunity in the gut (4). Recent research indicated that Foxp3+ T cells could repress inflammation, IgA production, and cause diversification of gut microbiota, forming a symbiotic regulatory loop (5).

Clinical symptoms of ulcerative colitis patients could be relieved to some extent with several treatments such as administration of antibiotic, but these clinical symptoms readily recur and side effects may occur, and most of available treatments have remission rates of less than 50% (6). Polysaccharides from mushroom were found to have positive effects on colonic inflammation by modifying gut microbiota in the colon, which is implicated in the maturation and education of host immune responses, protection against enteric pathogen proliferation (7, 8).

*Tremella fuciformis* is an edible medicinal mushroom, which has been widely cultivated. Its various bioactivities include immunomodulatory, anti-tumor, anti-oxidation, anti-aging, anti-inflammatory, repairing brain memory impairment, and lowering blood sugar and serum cholesterol levels (9, 10). The major bioactive components of *Tremella fuciformis* have been identified as polysaccharides of *Tremella fuciformis* (TPs), and under various experimental conditions different TPs fractions can be harvested, which gives TPs with a mixture of diverse polysaccharides a molecular weight ranging from 5.82 × 10^5^ Da to 3.74 × 10^6^ Da (10). TPs could reverse the effects of regulatory T cells on the proliferation and polarization of CD4+ T cells, down-regulate the IL-10 level, and reduce the mortality of septic mice (11). The application of TPs on IBD has not yet been explored. In this study, a new type of TPs could be isolated to ascertain the alleviation effect on DSS-induced mice and reveal the possible mechanisms related to modulation of the gut microbiome.

## Materials and Methods

### Preparation of TPs

TPs were extracted with boiling water in a solid-liquid ratio of 1:20 (w/v) for 6 h. After being filtered and centrifuged, three times the volume of ethanol was added to obtain a precipitate, and the precipitate was deproteinated using the Sevage method (12). The deproteinated part was extensively dialyzed against distilled water and then applied to a 60 mm × 240 m column of DEAE-cellulose (Sigma, USA) at a flow rate of 3.0 mL/min. The column was then eluted with distilled water, 50 mM NaCl, 150 mM NaCl, and 1M NaCl at 10 mM/min, successively, and the part eluted by 50 mM NaCl was collected and lyophilized: this was defined as the TPs for further study.

### Characterization of TPs

Molecular weights of TPs were determined by gel permeation chromatography (Wyatt, USA) on a Shodex OHpak SB column. The column was operated at 40°C with a flow rate of 1 mL/min. Monosaccharide composition of TPs was analyzed by liquid chromatography (Agilent 1200, USA) with a SHISEIDO C18 column. The column was operated at room temperature with a flow rate of 1 mL/min. The saccharides related to TPs were investigated by hydrolysis with dilute sulfuric acid according to the standard method of the National Renewable Energy Laboratory (CO, USA), with calibration applying a standard solution of rhamnose, arabinose, xylose, mannose, glucose, fucose, galactose, galacturonic acid, glucuronic acid, and ribose. The FT-IR spectra of TPs were collected with an FT-IR microscope (Model iN 10, Thermo Nicolet Corp.; Madison, WI, USA) equipped with a liquid nitrogen-cooled MCT detector (13).

### Mice and Treatment

Thirty female C57BL/6 mice (aged 6-7 weeks, mass 17.21 ± 0.27 g) were purchased from Chengdu Dossy Experimental Animals Co., Ltd (Chengdu, China). All mice were housed in specific pathogen-free environment in a standard 12 h light-dark cycle at 25°C and had *ad libitum* access to food and water. All mice were allocated to one of four groups (*n* = 6 per group): (1) Control group (received the standard diet); (2) DSS challenge group (received the standard diet); (3) DSS challenge + 200 mg/kg TPs (LTPs group; received the standard diet plus 200 mg/kg TPs); (4) DSS challenge + 300 mg/kg TPs (HTPs group; received the standard diet plus 300 mg/kg TPs). The treatment process lasted for two weeks. Mice were orally treated with TPs or normal saline (Control and DSS groups) once daily for 14 days, starting 7 days before the induction of colitis. Acute colitis was induced by 3.5% DSS given in distilled water for days 8-14 except to those in the control group. At day15, mice were sacrificed to yield samples for subsequent experiments.

### Serum Biochemical Analysis

Blood samples were collected from the coeliac artery, and then centrifuged at 2000*g* for 15 min to harvest serum for analysis. The D-lactate concentration and diamine oxidase (DAO) activity in serum were determined with commercial assay kits H263 and A088-1 from Nanjing Jiancheng Bioengineering Institute (Nanjing, China), respectively. 

### Bodyweight, Colon Length, Myeloperoxidase Activity, and Histological Analysis

After anesthesia the bodyweight of mice was recorded. Once the mice were sacrificed, the length of the colon was measured, and samples were processed for the following analysis. Colonic samples were homogenized in phosphate-buffered saline (PBS) buffer (pH 7.4) and centrifuged at 14,000*g* for 15 min at 4°C, and the supernatant was used for MPO activity tested with myeloperoxidase assay kit (Nanjing Jiancheng Bioengineering Institute, Nanjing, China) according to the manufacturer’s instructions. The colon tissue specimens were fixed with 10% neutral buffered formalin at room temperature, dehydrated in a graded ethanol series, and embedded in paraffin. Tissue sections were stained with hematoxylin and eosin (H & E) to identify histological change. Images were acquired using a microscope (Leica Microsystems, Wetzlar, Germany), and the villus height and crypt depth were measured according to the stained images with software Image-Pro Plus 6.0. The histological analysis was performed by an independent researcher in a single-blind manner (14). 

### Determinations of Intestinal Cytokine and Nuclear Receptor PPAR-γ Concentrations

After mice were sacrificed, colonic tissue was collected, weighed, and immediately preserved with liquid nitrogen. We thawed the specimens before testing and kept them at 2 to 8°C. We homogenized the specimens on ice with normal saline in the ratio of 1:9 (w/v) using a homogenizer. The homogenates were centrifuged at 3000*g* for 15 min at 4 °C, and the supernatant was harvested for subsequent analysis. The concentrations of different cytokines (IFN-γ, IL-1β, IL-6, TGF-β, IL-10, and TNF-α) in the colonic tissue supernatant were quantified using enzyme-linked immunosorbent assay (ELISA) kits (Shanghai ZCI Bioscience Co., Ltd, Shanghai, China) according to the manufacturer’s instructions. The PPAR-γ concentration was determined with a ZC-38225 Mouse PPAR-γ ELISA Kit (Shanghai Zhuo Cai Technology Co., Ltd, Shanghai, China) referring to the manufacturer’s instructions. 

### Quantification of Immune Cells in the Colonic Mucosa and IgA-Coated Bacteria in Intestinal Contents

Flow cytometric analysis of IgA-coated bacteria was implemented as described elsewhere (5) with some modifications. Intestinal contents were placed in a 1.5 mL tube and washed with 200 μL PBS. The supernatant was collected and passed through cell strainer, and then centrifuged at 500*g* for 5 min. The supernatant was discarded, and the precipitate was resuspended with 100 μL PBS for later use. FITC labeled anti-mouse IgA (C10-1) from BD Biosciences (5μL) was added to each tube containing the intestinal content specimens. These samples were protected from light at 4°C for 30 min, washed twice with 1 mL PBS, centrifuged at 500*g* for 5 min, the supernatant was discarded, and the cells were resuspended with 500 μL PBS. These samples were analyzed by flow cytometry.

Lymph node cells were collected from mesentery and analyzed for Foxp3+ Tregs, which was conducted with reference to Pei et al. (15). Lymph node cells were first stained with the following antibodies (BioLegend, CA): FITC-conjugated mAb to CD4 (RM4-5) and Alexa Fluor^®^ 647-conjugated mAb to CD25 (PC61). After fixation, intracellular staining with antibody of PE-conjugated mAb to Foxp3 (150D) was performed (BioLegend, CA). Data were acquired on a flow cytometry analysis (CytoFLEX, Beckman Coulter, USA). Except for this group, the lymph node samples were divided into another four groups, including a Blank group (unstained), CD4 single staining group, CD25 single staining group, and a Foxp3 single staining group.

### Gut Microbiota Analysis

Samples of intestinal contents from control group, DSS challenge group, and HTPs group were collected. Total genome DNA from intestinal content was extracted by using the CTAB/SDS method and was subjected to 16S amplifications using primers designed to incorporate both the Illumina adapters and a sample barcode sequence, allowing directional sequencing that covers variable region V4 (Primers: 515 F [GTGCCAGCMGCCGCGGTAA] and 806R [GGACTACHVGGGTWTCTAAT]. PCR reactions were performed with Phusion^®^ High-Fidelity PCR Master Mix (New England Biolabs, USA). Sequencing libraries were generated using Ion Plus Fragment Library Kit 48 rxns (Thermo Scientific, USA) following manufacturer’s recommendations. The library was sequenced on an Ion S5™ XL platform and 400 bp/600 bp single-end reads were generated. All the results were based on sequenced reads and operational taxonomic units (OTUs). Analysis of 16S sequences was undertaken by UPARSE software (v7.0.1001), and sequences with ≥ 97% similarity were assigned to the same OTUs. The Silva Database (https://www.arb-silva.de/) was used based on the Mothur algorithm to annotate taxonomic information. Alpha diversity including observed-species, Chao1, Shannon, Simpson, ACE, good coverage, and beta diversity were both calculated with QIIME (Version1.7.0) and displayed with R software (Version 2.15.3). All 16S rRNA sequencing data were saved in the National Center for Biotechnology Information and can be accessed in the Short Read Archive under accession number PRJNA679459 (http://www.ncbi.nlm.nih.gov/bioproject/679459).

### Measurement of Fecal Metabolomics

Samples of intestinal contents of control group, DSS challenge group and HTPs group specimens were collected. Each sample was individually ground with liquid nitrogen and the homogenate was resuspended with pre-chilled 80% methanol and 0.1% formic acid. The samples were centrifuged, and supernatant was diluted to final concentration containing 60% methanol by LC-MS grade water. The samples were subsequently transferred to a fresh Eppendorf tube with 0.22-μm filter, then centrifuged. Finally, the filtrate was injected into the LC-MS/MS system for analysis.

LC-MS/MS analyses were conducted using a Vanquish UHPLC system (Thermo Fisher) coupled with an Orbitrap Q Exactive™ series mass spectrometer (Thermo Fisher). Samples were injected onto an Hypersil Gold column (100 mm × 2.1 mm, 1.9 μm) using a 16-min linear gradient at a flow rate of 0.2mL/min. The eluents for the positive polarity mode were eluent A (0.1% FA in water) and eluent B (methanol). The eluents for the negative polarity mode were eluent A (5 mM ammonium acetate, pH 9.0) and eluent B (Methanol). The solvent gradient was set as follows: 2% B, 1.5 min; 2-100% B, 12.0 min; 100% B, 14.0 min; 100-2% B, 14.1 min; 2% B, 16 min. Q Exactive mass series spectrometer was operated in positive/negative polarity mode with spray voltage of 3.2 kV, capillary temperature of 320°C, sheath gas flow rate of 35 arb and auxiliary gas flow rate of 10 arb. The metabolites were annotated using the KEGG database (http://www.genome.jp/kegg/), HMDB database(http://www.hmdb.ca/) and LIPID MAPS^®^ Structure Database (LMSD) (http://www.lipidmaps.org/). Principal components analysis (PCA) and partial least squares discriminant analysis (PLS‐DA) were performed at MetaX. Univariate analysis (*t*-test) was conducted to determine the statistical significance (*P*-value). Those metabolites with VIP > 1, *P*-value < 0.05, and fold-change ≥ 2 or fold-change ≤ 0.5 were considered differential metabolites. 

### Data Analysis

Statistical analysis was carried out using one-way analysis of variance (ANOVA) followed by Tukey test using SPSS 22.0 software (IBM, Chicago, IL, USA). Data are presented as mean ± standard deviation (SD) or as a scatter plot. The correlations between relative abundances of OTUs and microbial metabolites were analyzed by Spearman’s correlation (SPSS 22, IBM): *P* < 0.05 was considered statistically significant.

## Results and Discussion

### Molecular Weight, Monosaccharide Composition, and Infrared Spectral Analysis of TPs

Molecular weights of TPs harvested from HP-GPC detection are listed in **Table 1**. *M*_w_ and *M*_n_ values of TPs were 288.6 and 268.8 kDa, respectively. The *D* value (*M*_w_/*M*_n_) was 1.074, suggesting that the TPs were relatively pure. Molecular weights of TPs were mainly distributed between 246 and 305 kDa (**Table 2**). The monosaccharide components of TPs are displayed in **Table 3**. The main monosaccharide sector comprised mannose followed by glucuronic acid, xylose, fucose, glucose, galactose, arabinose, rhamnose, and ribose.

**Table 1:** Molecular weights.

Mw/Mn	Mz/Mn	Mn, kDa	Mp, kDa	Mw, kDa	Mz, kDa
1.074	1.177	268.8	309.0	286.6	316.4

**Table 2:** Molecular weight distribution.

Molecular weight distribution	154000.0-246000.0 g/mol	246000.0-305000.0 g/mol		305000.0-400000.0 g/mol	400000.0-770484.0 g/mol
%	15.2	42.0	36.0		6.8

**Table 3:** Monosaccharide composition.

Monosaccharide composition (mg/kg)								
Mannose	Ribose	Rhamnose	Glucuronic acid	Glucose	Galactose	Xylose	Arabinose	Fucose
91120.57	337.73	623.67	29067.09	2086.89	1656.21	25035.97	1540.07	21388.01

**Figure 1:**
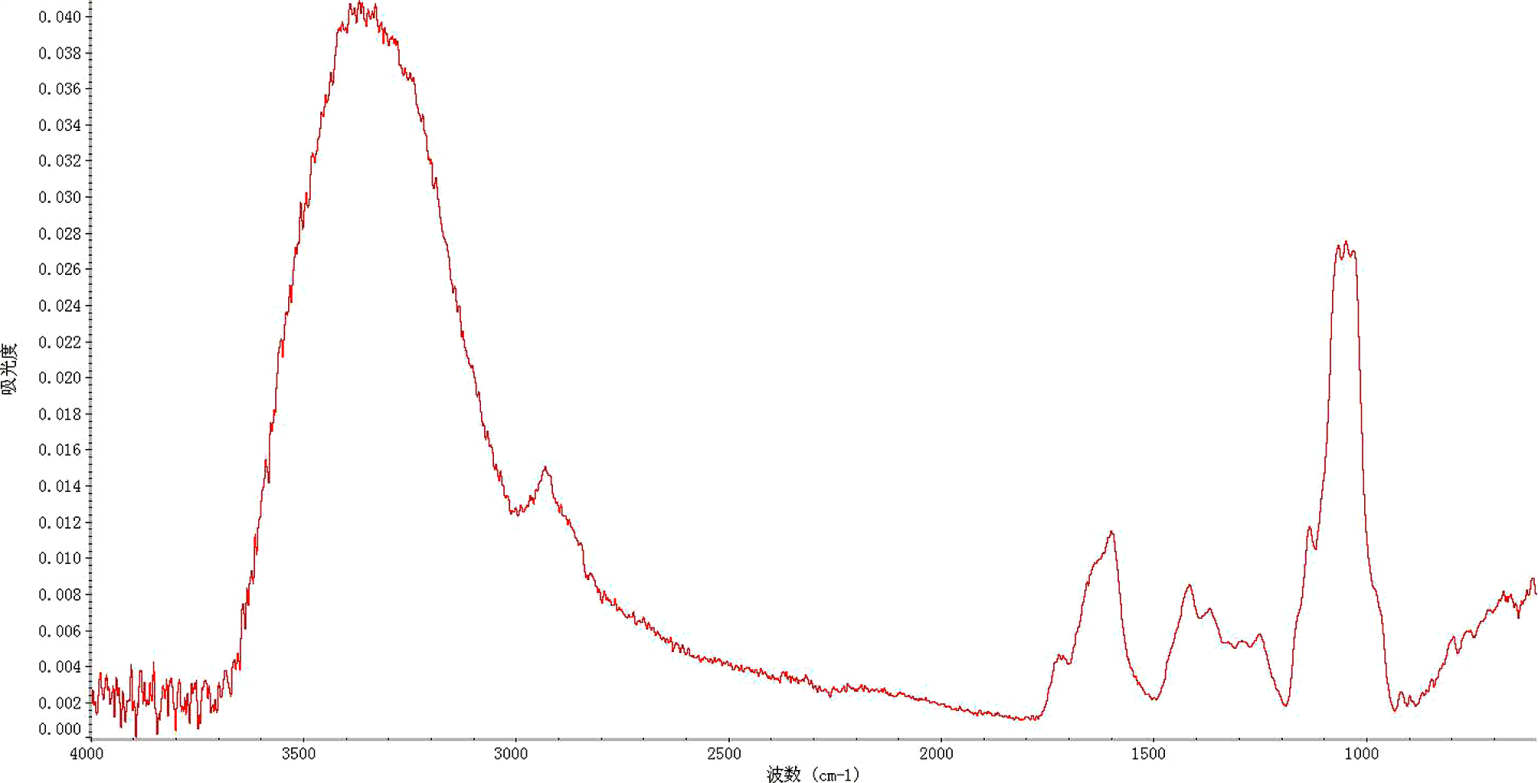
FT-IR spectrum of TPs.

Infrared spectroscopy reflected the characteristic absorption peak of polysaccharides used when analyzing the structure of these TPs. **Figure 1** shows that absorption peaks at 3300-3000 cm^-1^, 1675-1640 cm^-1^, and 1000-675 cm^-1^ indicated the presence of double bonds. The presence of a formyl group was evinced by the absorption peaks at 1300-1000 cm^-1^ and 769-659 cm^-1^. The absorption peaks at 3000 cm^-1^ and 1600 cm^-1^ suggested the presence of an aromatic ring. Strong absorption of 3500 to 3200 cm^-1^ and 1300 to 1000 cm^-1^ showed the presence of an alcohol hydroxyl group. According to infrared spectroscopy analysis, there was 78.24% similarity between TPs and hemicellulose. 

### TPs Ameliorated Clinical Symptoms in the DSS-Induced Colitis Mice Model

**Figure 2:**
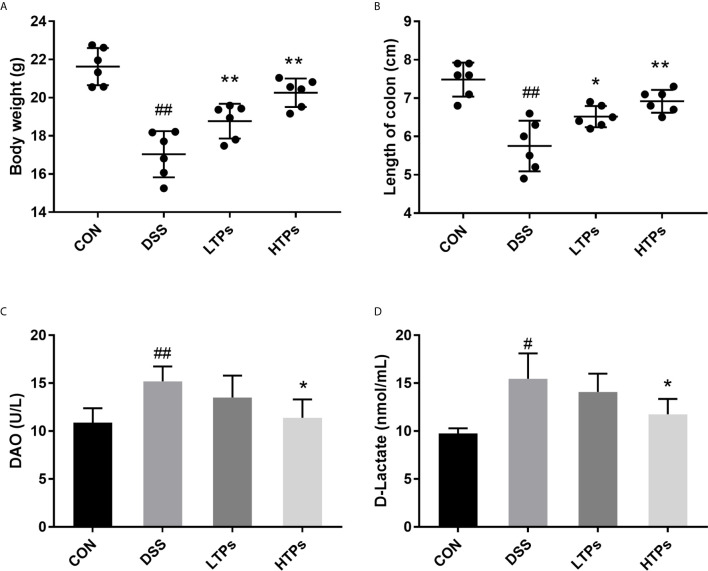
TPs ameliorated DSS-induced clinical symptoms in mice. **(A)** Bodyweights of mice were measured after the experiment. **(B)** Length of colon. **(C)** DAO activity in serum. **(D)** D-lactate concentration in serum. CON, Control group; DSS, DSS challenge group; LTPs, DSS challenge + 200 mg/kg TPs; HTPs, DSS challenge +300 mg/kg TPs. All values are represented as mean ± SD (*n* = 6). ^#^ *p* < 0.05, ^##^ *p <* 0.01 *v*. normal group. *p < 0.05, **p < 0.01 *v*. control group.

**Figure 3:**
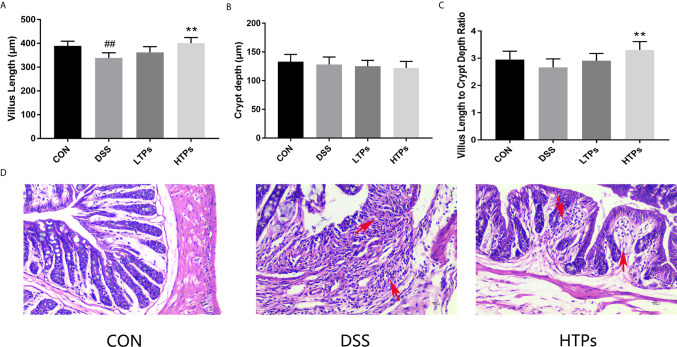
Effects of HTPs on colonic morphology in DSS-challenged mice. **(A)** Villus height. **(B)** Crypt depth. **(C)** Villus length to crypt depth ratio (VCR). **(D)** Representative images of H & E staining of the colon tissue (magnification 400×). CON, control group; DSS, DSS challenge group; LTPs, DSS challenge + 200 mg/kg TPs; HTPs, DSS challenge +300 mg/kg TPs. All values are represented as mean ± SD (*n* = 6). ^##^ *p* < 0.01 *v*. normal group. **p < 0.01 *v*. control group.

The typical symptoms and pathological changes of colitis involve bodyweight loss, shortening of colon, serum DAO activity, D-lactate concentration increase, hyperplasia of crypts, *etc.* (16–18). As shown in **Figures 2A, B**, a significant decrease of bodyweight and shortening of colon could be found after DSS treatment. TPs improved weight and length, with HTPs being the most beneficial. Intestinal epithelial barrier deficiencies along with abnormal intestinal permeability are crucial pathogenic factors in IBD (19). In our study, HTPs treatment reduce DAO activity and D-lactate concentration in serum which reflects the intestinal barrier integrity compared with DSS group (**Figure 2**). Villus height, crypt depth, and villus length to crypt depth ratio (VCR) reflect gross intestinal morphology (20): as shown in **Figure 3**, HTPs treatment prevented loss of villus height and VCR of colon, and largely ameliorated colon inflammatory symptoms, including relative intact surface epithelium, less inflammatory cell infiltration, and mild submucosal oedema. Thus, TPs treatments significantly reduced the abnormal histopathological changes in the colonic tissues. These results indicated that pre-treatment with TPs could effectively inhibit the severity of colitis in DSS-treated mice.

### Effects of TPs on Inflammation in DSS-Induced Colitis Mice

**Figure 4:**
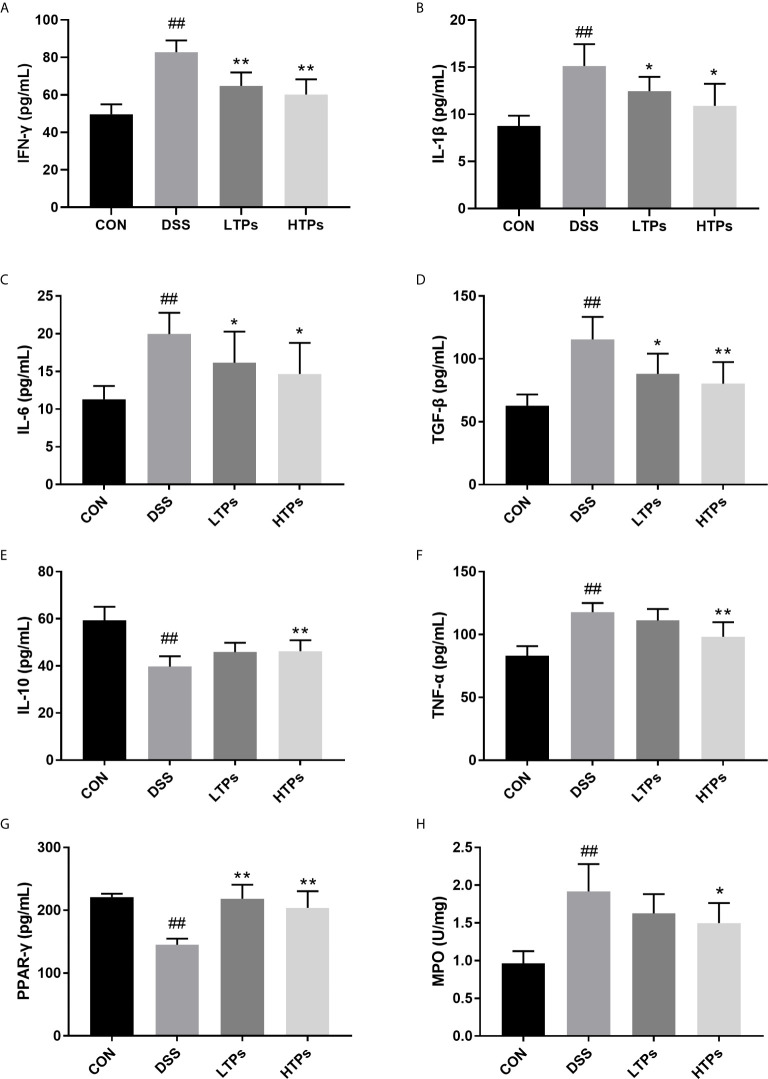
Quantification of the concentrations of IFN-γ **(A)**, IL-1β **(B)**, IL-6 **(C)**, TGF-β **(D)**, IL-10 **(E)**, TNF-α **(F)**, PPAR-γ **(G)** and MPO **(H)** activity in colon. All values are represented as mean ± SD (*n* = 6). CON, Control group; DSS, DSS challenge group; LTPs, DSS challenge + 200 mg/kg TPs; HTPs, DSS challenge +300 mg/kg TPs. ^#^ *p *< 0.05, ^##^ *p* < 0.01 *v*. normal group. *p < 0.05, **p < 0.01 *v*. control group.

Colitis is associated with chronic inflammation resulting from loss of IL-10 and increase of pro-inflammatory cytokines such as IFN-γ, IL-1β, IL-6, TGF-β, and TNF-α (21, 22). PPAR-γ is a member of a superfamily of nuclear receptors, which possess crucial anti-inflammatory activity, and can inhibit the expression of IL-1β and TNF-α (23). The values of pro-inflammatory cytokines were remarkably increased in the DSS group while anti-inflammatory cytokines were notably decreased compared with those in the control group (**Figure 4**). Treatment with TPs significantly enhanced anti-inflammatory cytokines IL-10 and lowered pro-inflammatory cytokines (IFN-γ, IL-1β, IL-6, TGF-β, and TNF-α). Additionally, the HTPs had better anti-inflammatory effect. Both low-dose and high-dose TPs treatment notably elevated PPAR-γ concentration in colonic tissue. It has been shown that promoting PPAR-γ activation could protect mice from DSS-induced colitis (24). This result offered further proof of the effects of TPs in alleviating DSS-induced colitis. The activity of MPO was an index of neutrophil infiltration and inflammation, and the level of MPO activity reflected the level of colonic inflammation (25). MPO level in DSS-treated colon tissue was found to increase significantly (**Figure 4H**). The HTPs group decreased the MPO level, indicating that HTPs (300 mg/kg) alleviated inflammation in the colon.

**Figure 5:**
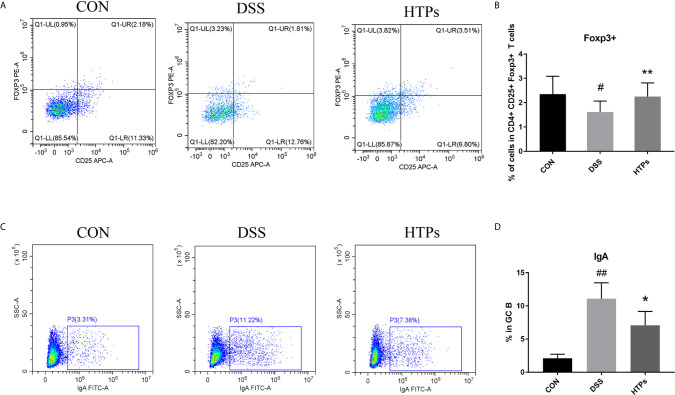
Flow cytometry analysis of the populations of Treg (CD4+CD25+Foxp3+) and IgAs from C57BL/6 mice. Representative dot plots from mesenteric lymph nodes and intestinal contents. Treg (CD4+CD25+Foxp3+) and IgAs in **(A, C)** respectively. Relative expression of Treg (CD4+CD25+Foxp3+) and IgAs in **(B, D)**. CON, Control group; DSS, DSS challenge group; LTPs, DSS challenge + 200 mg/kg TPs; HTPs, DSS challenge +300 mg/kg TPs. All values are represented as mean ± SD (*n *= 6). ^#^ *p* < 0.05, ^##^ *p* < 0.01 *v*. normal group. *p < 0.05, **p < 0.01 *v*. control group.

It has been shown that Foxp3+T cells regulated the expansion of naïve CD4+T cells and their production of pro-inflammatory cytokines. The production of pro-inflammatory cytokines such as TNF-α and IFN-γ decreased while the level of anti-inflammatory cytokine IL-10 increased in the presence of Foxp3+T cells (26). By flow cytometry analysis, the proportion of Treg cells (CD4+CD25+FoxP3+) in the mesenteric lymph nodes of the HTPs-treated group was significantly higher than in the DSS group (**Figures 5A, B** **)**, which shows that HTPs treatment promoted Foxp3+T cells. IgAs are mostly produced in mucus membranes. Leakage of serum into the gut at sites of inflammation could yield a higher percentage of IgA-coated bacteria in IBD patients (27, 28). In our study, the DSS treatment increased IgA-coated bacteria proportion in the gut as shown in **Figures 5C, D**; however, feeding with HTPs notably decreased the proportional increase therein. IgA played an important role in the balance of gut bacterial communities (5, 27). HTPs treatment affected the expansion of Foxp3+T cells and IgA production to reduce the gut inflammatory status and adjusted the balance of the gut microbiome. 

### Gut Microbiota Analysis

**Figure 6:**
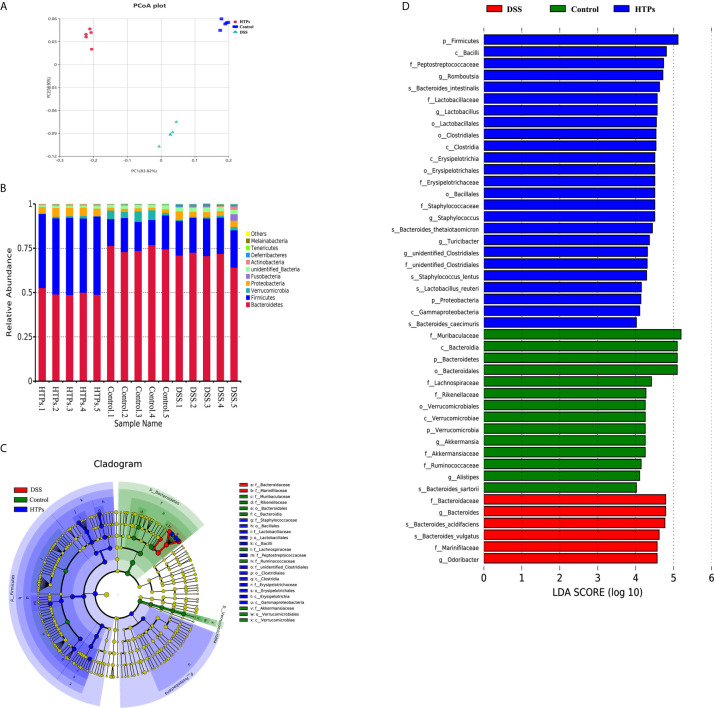
**(A)** Beta diversity parameters measured with PCoA plot. Each point designates an individual sample, and the points of different colors represent various treatments. The distance between different points demonstrates the similarity or differences of the microbial community structure. **(B)** Microbial composition at phylum levels in HTPs, control and DSS groups. Linear discriminant analysis effect size (LEfSe) analysis of microbiota in HTPs, control and DSS groups: **(C)** taxonomic cladogram demonstrating highly abundant taxa across various treatments. **(D)** taxa that meet an LDA score threshold of > 4 (*n* = 5).

Perturbations in the intestinal microbiome induced host physiology altering by affecting homeostasis, barrier function, innate and adaptive immune responses, and metabolism (29, 30). Illumina Miseq (16S rRNA gene) was employed to characterize the overall pattern of gut microbiota community in control, DSS- and HTPs-treated mice. In our study, alpha and beta diversity indices were analyzed to illuminate bacterial richness, diversity, and structural differences in each group (**Table 4**, **Figure 6A**). According to Shannon and Simpson indices, DSS treatment decreased gut community diversity to a significant extent. With HTPs treatment, the Simpson indices increased again. Referring to Chao 1 and Ace indices, DSS treatment induced no significant change, but HTPs treatment decreased community richness compared with the specimens in the DSS group. Analysis of beta diversity with PCoA (**Figure 6A**) showed that gut microbiota in control, DSS and HTPs groups were significantly different in terms of the cumulative contributions made by the first two principal components which accounted for 96.72% of all species herein. As a result, DSS and HTPs treatment caused significant changes in the composition of gut microbiome.

**Table 4:** Alpha diversity parameters assessed by Ace, Chao1, Shannon, and Simpson indices.

Item	Treatment		
	CON	DSS	HTPs
Shannon index	6.27 ± 0.07	5.71 ± 0.20^**^	5.72 ± 0.08
Simpson index	0.97 ± 0.00	0.94 ± 0.01^**^	0.96 ± 0.00^##^
Chao1 index	393.09 ± 30.64	371.89 ± 24.59	323.8794 ± 12.75^#^
ACE index	390.55 ± 23.95	374.08 ± 24.29	328.50 ± 13.87^#^

CON, Control group; DSS, DSS challenge group; HTPs, DSS challenge 300 mg/kg TPs.

**p < 0.01 v. the CON group. ^#^p < 0.05 or ^##^p < 0.01 v. the DSS group.

**Figure 7:**
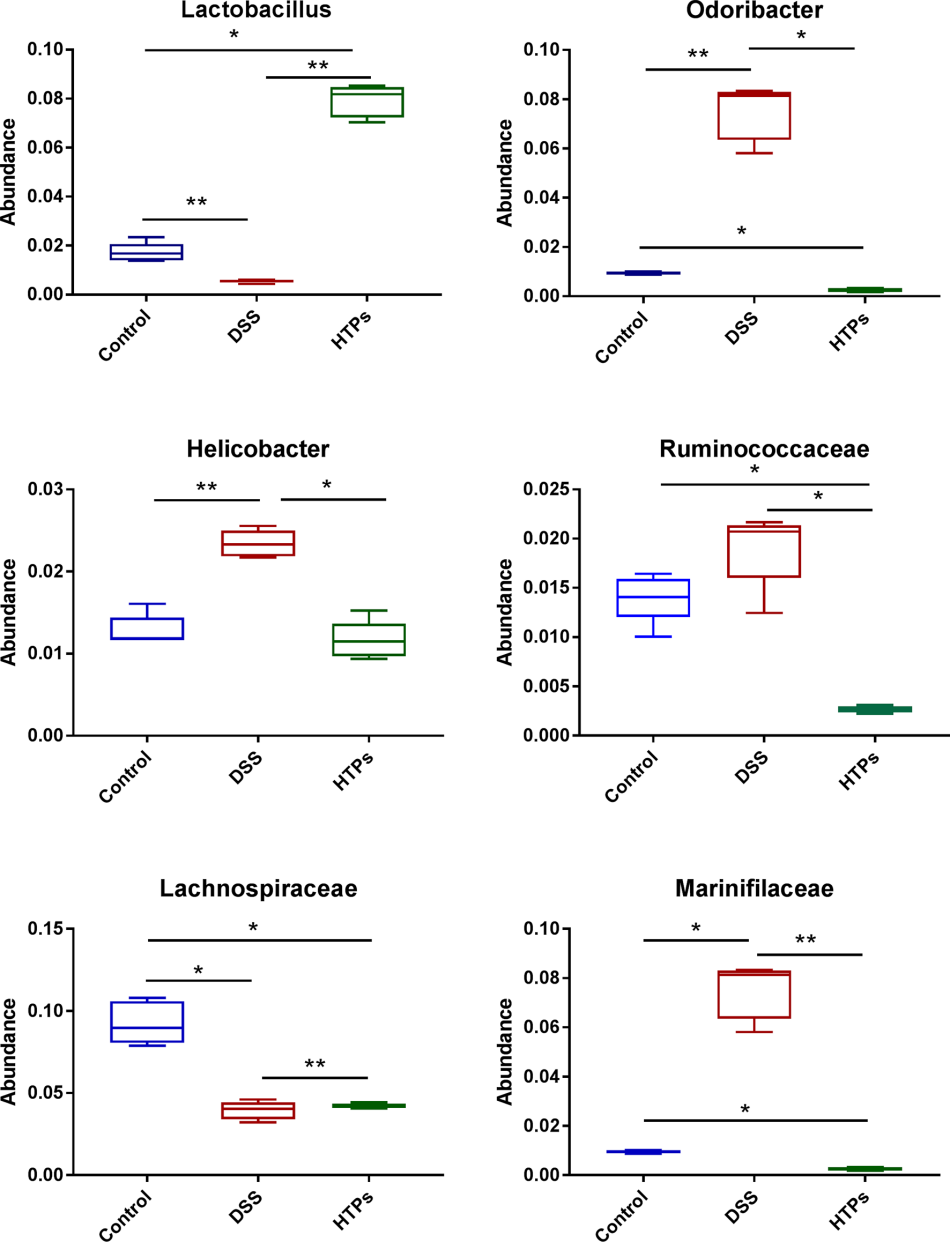
Relative abundances of Lactobacillus, Odoribacter, Helicobacter, Ruminococcaceae, Lactobacillaceae, and Marinifilaceae across each grouped microbiome. All values are represented as mean ± SD (*n *= 5).*p < 0.05, **p < 0.01.

**Figure 6B** illustrates the microbial composition at phylum level in HTPs, control, and DSS groups. Firmicutes and Bacteroidetes are important in adjusting absorption, energy transformation, and glucose metabolism which are parts of the body’s energy balance mechanism (31). The higher ratio of Firmicutes/Bacteroidetes in the HTPs group may have the potential ability to enhance the capacity to absorb calories from foods by gut microbes compared with control and DSS groups. HTPs treatment could notably alter the abundances of Lactobacillus, Odoribacter, Helicobacter, Ruminococcaceae, Lactobacillaceae, and Marinifilaceae to normal level compared with the control group (**Figure 7**). The abundances of Lactobacillaceae and Lactobacillus were significantly elevated after HTPs treatment compared with DSS treatment, which is consistent with Yanhui Han’s finding that strawberry restored Lactobacillus in colonic mice and Lactobacillus possessed protective immunoregulatory properties against colonic inflammation (32, 33). Lactobacillaceae generated formic acid, lactic acid, acetic acid, and ethanol by fermentation of glucose and pectin, and acetic acid was associated with improved energy and lipid metabolism (34). The significantly decreased abundance of Helicobacter in HTPs group mice compared with control group helped to restore healthy gut microbiome as infection with Helicobacter spp. has been reported to cause IBD in immunodeficient mice (35). Ruminococcaceae in the individuals who were suffering from chronic inflammatory bowel disease were found to be elevated, but HTPs helped to lower the abundance of Ruminococcaceae in the colon (36). Our results pertaining to Odoribacter conflict with earlier findings suggesting that Odoribacter is more abundant in healthy individuals, and the decrease of Odoribacter is related to host inflammation (37). Odoribacter is a producer of acetic acid, propionic acid, and butyric acid, and the reduction of Odoribacter resulted in a shortage of short-chain fatty acids (SCFA), which led to host intestinal inflammation (38); however, the analysis suggests a correlation between the microbiome and metabolites in our study (**Figure 9**), and Odoribacter has no significant relationship with SCFA. As the gut microbiome is diverse, Odoribacter occupies a small part thereof, which may contribute little in the present study: much remains to be explored with regard to this issue.

Cladogram and linear discriminant analysis (LDA) (**Figures 6C, D** **)** were used to demonstrate the hierarchy and abundance of gut microbiome between different groups. Firmicutes, Proteobacteria, Verrucomicrobia, and Bacteroidetes were the dominant phyla in all groups. LDA also showed the highest abundance of Firmicutes in the HTPs group which was in agreement with previous microbial composition analysis. The higher abundance of Bacteroides in DSS group in this study was consistent with reports that commensal Bacteroides play a potential role in proinflammation in colitic mice (39). Lactobacillus also exhibited a higher LDA score in the HTPs group. Generally, HTPs treatment modulated the perturbed gut flora in colitic mice to various degrees by increasing the abundances of Lactobacillus and Lactobacillaceae, and reducing the abundances of Odoribacter, Helicobacter, Ruminococcaceae, and Marinifilaceae. 

### Metabolite Analysis

**Figure 8:**
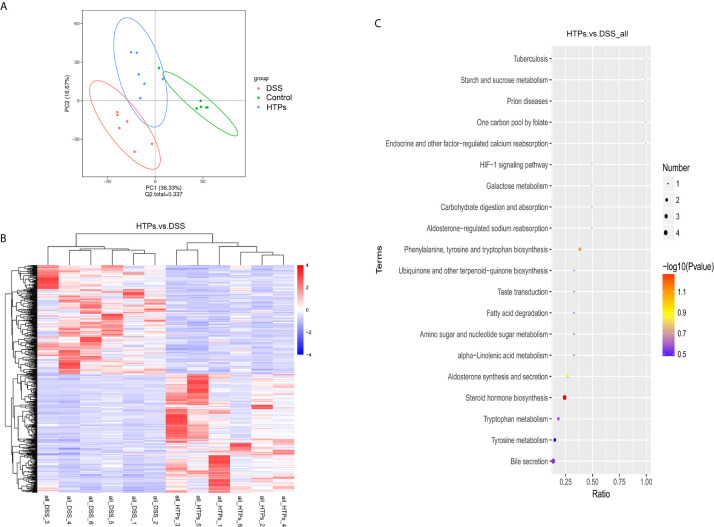
**(A)** Partial least squares discriminant analysis (PLS-DA) score plots comparing total fecal metabolites in HTPs, DSS and control groups. **(B)** Hierarchical clustering significantly changed metabolites of HTPs and control groups. VIP > 1.0, FC > 1.5, or FC < 0.667 and *P*-value < 0.05. **(C)** KEGG pathway enrichment of the significantly changed metabolites of HTPs and control groups. The *x*-coordinate is the ratio of the number of significantly changed metabolites in the corresponding metabolic pathways to the total number of metabolites in the identified pathway. The higher the value, the greater the enrichment of significantly changed metabolites in the pathway. The color of the dot represents the *P*-value of the hypergeometric test: the smaller the *P*-value, the more reliable and statistically significant the test. The size of the dot represents the number of significantly changed metabolites in the corresponding pathway. The bigger the dot, the greater the number of significantly changed metabolites in the pathway (*n* = 5).

**Figure 9:**
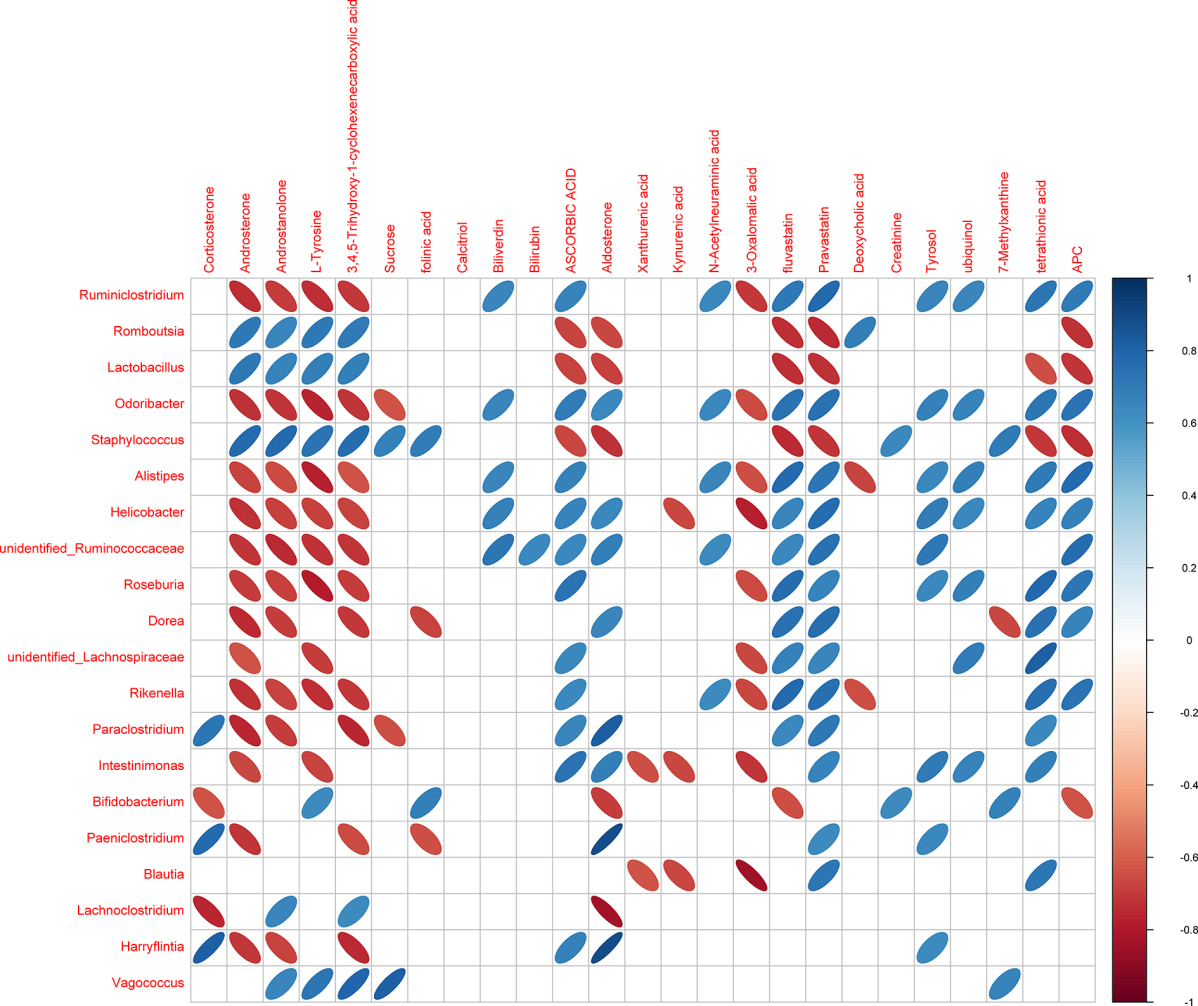
Heatmap of Spearman’s correlation between the microbiome and metabolites. The abscissa denotes the top-20 different metabolites and the ordinate is the top-10 different microbiomes at generic level in the HTPs and DSS groups. The color represents a significant correlation (*P* ≤ 0.05), and the color scale denotes Spearman’s correlation from blue (positive correlation) to red (negative correlation) (*n* = 5).

**Figure 10:**
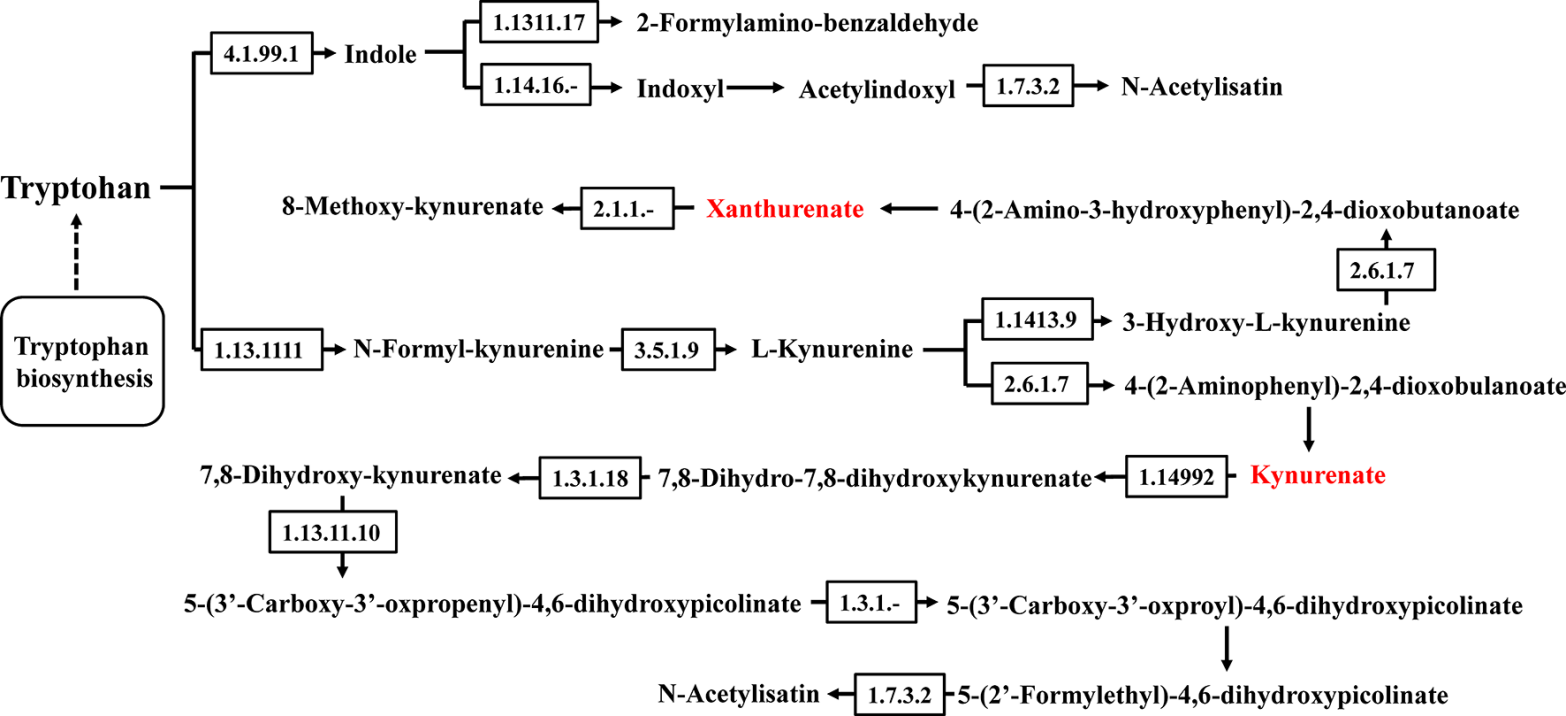
Tryptophan metabolism pathway.

The gut microbiota can offer energy and vitamins for the host by microbial metabolites and directly influence a variety of aspects of metazoan physiology (40). Microbial metabolites are crucial for host-microbial interactions and regulate host immune responses. LC-MS/MS was conducted to analyze the colonic digesta metabolic profiles: 3491 metabolites were detected and 454 of them were significantly changed because of HTPs exposure (**Supplementary Table 1**). Partial least squares discriminant analysis (PLS-DA) score plots showed significant changes in the composition of fecal metabolites of different groups. In this study, fecal metabolites in HTPs, control, and DSS groups were almost separated from each other in terms of their different compositions (**Figure 8A**). Clustering based on different metabolites implied that all samples were independent and unique in the metabolome dimensions, and 217 significantly changed metabolites were down-regulated and 237 were up-regulated (**Figure 8B**). Kyoto Encyclopedia of Genes and Genomes (KEGG) was adopted to analyze the top 20 enriched pathways of significantly different metabolic changes between DSS and HTPs groups (**Figure 8C**). The metabolic pathways of bile secretion, phenylalanine, tyrosine, and tryptophan biosynthesis, tyrosine metabolism, tryptophan metabolism, and steroid hormone biosynthesis, *etc.* were enriched. A significant elevation of L-tyrosine (Com_156_pos) was observed in HTPs group, and this increasement also occurred in fermentation supernatants of fecal inocula from healthy volunteers and edible mushroom *Pleurotus eryngii, Pleurotus ostreatus* and *Cyclocybe cylindracea* (*Basidiomycota*), which protected human colon adenocarcinoma Caco-2 against tert-butyl hydroperoxide (41). Referring to correlation analysis of significantly changed metabolites and microbiomes, L-tyrosine was elevated with the increased count of Lactobacillus and decreased counts of Odoribacter, Helicobacter, and Ruminococcaceae (**Figure 9**). We wonder whether HTPs may increase L-tyrosine levels through restoring Lactobacillus, Odoribacter, and Helicobacter. The reduction of intestinal tryptophan catabolites may have effects on the severity of IBD (42). The tryptophan metabolites of HTPs group, Xanthurenic acid (Com_5405_pos), and Kynurenic acid (Com_763_pos) were up-regulated compared with those in the DSS group (**Figure 10**). Thus, HTPs may protect the colon from inflammation through elevating the level of tryptophan catabolites. Due to indole derivatives being an indispensable part of the tryptophan catabolites generated by the gut microbiota, they affect host physiology in numerous ways, such as decreasing intestinal permeability, altering innate and adaptive immune responses, suppressing appetite, and inducing anti-oxidative and anti-inflammatory effects (43). In our study, indole derivatives detected in HTPs fecal matter including 5-methoxy-3-indoleaceate (Com_9624_pos), 5-hydroxyindole (Com_4464_pos), methyl (3-hydroxy-2-oxo-2,3-dihydro-1H-indol-3-yl) acetate (Com_12338_pos), 5-hydroxyindole-3-acetic acid (Com_317_pos), and 5-hydroxyindoleacetic acid (Com_972_neg), and these were all significantly increased compared with levels found in the DSS group. It is found that *Lactobacillus* strains metabolizing tryptophan could attenuate intestinal inflammation *via* aryl hydrocarbon receptor activation, especially, *Lactobacillus reuteri* which actives the aryl hydrocarbon receptor by way of indole derivatives to regulate intraepithelial CD4+ T helper cells in their transformation to immunoregulatory T cells (CD4+ CD8αα double-positive intraepithelial lymphocytes). In our study, the prominent increase in *Lactobacillus* in the HTPs group, whether it induced Foxp3+ T cell expansion by elevating tryptophan metabolites, required further proof. Bile acids were metabolized in the intestine by the gut microbiota, and the deconjugated primary bile acids were metabolized through gut microbial 7-dehydroxylation into secondary bile acids (lithocholic acid and deoxycholic acid) in the colon (44). In our study, the secondary bile acid (deoxycholic acid, Com_32_neg) was detected at significantly increased level compared with DSS group specimens in microbial metabolites. The analysis of the correlation between the microbiome and metabolites (**Figure 9**) indicated that deoxycholic acid was positively correlated with *Romboutsia*, which belongs to the Clostridium species. It is found that bacteria with capability to produce secondary bile acids were identified in Clostridium as belonging to the Firmicutes phylum (45), showing that Romboutsia may stimulate deoxycholic acid generation in the intestine to modify bile acid metabolism. Thus, HTPs may adjust microbial metabolites in colitic mice to a normal level through influencing tyrosine biosynthesis, tryptophan metabolism, and bile acid metabolism.

## Conclusion

HTPs treatment exerted a profound influence on colitis in mice by targeting host factors and microorganisms, including microbial physiology and metabolites. With HTPs treatment the typical symptoms and pathological changes of colitis were in remission. This effect was closely associated with immunoregulation of the host which as evinced by the fact that Foxp3+ T cell expansion and IgA-coated bacteria reduction, augment anti-inflammatory cytokines and decrease pro-inflammatory cytokines in colitic mice. HTPs treatment led to gut flora compositional changes and modulated microbial metabolites in HTPs group in a similar manner to that in the control group. Results of gut microbiota and microbiota metabolism analyses showed that HTPs improved the metabolites and metabolic pathways related to the change in inflammation caused by colitis and had an effective influence on intervention in DSS-induced colitis. This is the first study to ascertain the underlying mechanism of TPs in colitis through multi-path analysis of gut microbiota and metabolites. It offers a deeper perception of the colonic inflammation-inhibiting function of TPs and provides a new orientation for the application of TPs-related products.

## Data Availability Statement

The datasets presented in this study can be found in online repositories. The names of the repository/repositories and accession number(s) can be found below: https://www.ncbi.nlm.nih.gov/, PRJNA679459.

## Ethics Statement

The animal study was reviewed and approved by the guidelines of the Committee on Care and Use of Laboratory Animals of the Institute of Medicinal Plant Development, Chinese Academy of Medical Sciences.

## Author Contributions

WP obtained financial support and conceived this study. YX designed the animal experiment and wrote the manuscript. LX, ZZ, and WZ performed the animal experiment, generated the tissue samples and prepared testing samples. XH, JT, and JZ analyzed the data. All authors contributed to the article and approved the submitted version.

## Funding

The National Modern Agro-industry Technology Research System (CARS-24) and the Science and Technology Project of Sichuan Province(2017NZ0006).

## Conflict of Interest

The authors declare that the research was conducted in the absence of any commercial or financial relationships that could be construed as a potential conflict of interest.
