# Supplementary material for: Tremella fuciformis Polysaccharides Inhibited Colonic Inflammation in Dextran Sulfate Sodium-Treated Mice via Foxp3+ T Cells, Gut Microbiota, and Bacterial Metabolites
Source: Front Immunol. 2021 Apr 1;12:648162. doi: 10.3389/fimmu.2021.648162 (PMC8049506; doi:10.3389/fimmu.2021.648162)
Supplement: Supplementary file 1 [file Table_1.docx]

**Supplementary Table 1**: Significantly changed metabolites in DSS and HTPs group

| **ID** | **Name_des** | **Formula** | **Molecular Weight** | **FC** | **log2FC** | **Pvalue** | **VIP** |
| --- | --- | --- | --- | --- | --- | --- | --- |
| Com_19184_pos | butaperazine | C24 H31 N3 O S | 409.2204 | 0.411256 | -1.28189 | 4.43E-06 | 1.272263 |
| Com_3817_neg | 20-hydroxy-leukotriene E4 | C23 H37 N O6 S | 455.2343 | 0.163989 | -2.60833 | 3.18E-05 | 2.685142 |
| Com_2628_pos | Urothion | C11 H11 N5 O3 S2 | 325.0296 | 0.351336 | -1.50908 | 7.06E-05 | 1.504248 |
| Com_14191_neg | Phenothiazine, 10-[3-(dimethylamino)propyl]-, 5-oxide | C17 H20 N2 O S | 300.1302 | 0.244293 | -2.03332 | 9.03E-05 | 2.01056 |
| Com_1504_neg | Epelsiban | C30 H38 N4 O4 | 518.2914 | 0.445819 | -1.16547 | 9.84E-05 | 1.172425 |
| Com_15379_pos | L-(+)-Cysteine | C3 H7 N O2 S | 121.02 | 0.242769 | -2.04234 | 0.000105 | 2.089299 |
| Com_869_neg | (10E,15Z)-9,12,13-Trihydroxy-10,15-octadecadienoic acid | C18 H32 O5 | 328.2252 | 2.618948 | 1.388987 | 0.000225 | 1.350478 |
| Com_733_neg | apigetrin | C21 H20 O10 | 432.1059 | 31.33348 | 4.969633 | 0.000228 | 4.975347 |
| Com_1213_pos | albocycline | C18 H28 O4 | 308.198 | 2.988526 | 1.579434 | 0.000254 | 1.573876 |
| Com_1079_neg | Aurantio-obtusin beta-D-glucoside | C23 H24 O12 | 492.1269 | 34.00389 | 5.087628 | 0.000259 | 5.439039 |
| Com_947_pos | Genistin | C21 H20 O10 | 432.1049 | 38.23056 | 5.256654 | 0.000286 | 5.568427 |
| Com_3176_pos | 15-Deoxy-Δ12,14-prostaglandin A1 | C20 H30 O3 | 318.2186 | 0.236788 | -2.07833 | 0.000292 | 2.099827 |
| Com_7135_pos | D-Arginyl-L-arginyl-D-isoleucine | C18 H37 N9 O4 | 443.2987 | 0.258551 | -1.95148 | 0.0003 | 1.852099 |
| Com_392_neg | his-asp | C10 H14 N4 O5 | 270.0951 | 5.569617 | 2.477578 | 0.000324 | 2.504772 |
| Com_9624_pos | 5-Methoxy-3-indoleaceate | C11 H11 N O3 | 205.0737 | 10.92869 | 3.450049 | 0.000339 | 3.130597 |
| Com_2313_pos | daphnoline | C35 H36 N2 O6 | 580.2582 | 0.153886 | -2.70007 | 0.000348 | 2.577396 |
| Com_6242_neg | Amphibine A | C33 H43 N5 O4 | 573.3342 | 0.210906 | -2.24533 | 0.000429 | 2.223395 |
| Com_10567_pos | SL4860000 | C13 H20 O2 | 208.1462 | 2.257713 | 1.174862 | 0.000457 | 1.215712 |
| Com_2355_neg | 3-oxopalmitic acid | C16 H30 O3 | 270.2194 | 2.571451 | 1.362583 | 0.000482 | 1.315392 |
| Com_1263_pos | Daidzin | C21 H20 O9 | 416.1101 | 43.39203 | 5.439358 | 0.000521 | 5.60421 |
| Com_4842_pos | Glycitin | C22 H22 O10 | 446.1207 | 32.3629 | 5.016269 | 0.000537 | 4.789756 |
| Com_347_neg | Myristoleic acid | C14 H26 O2 | 226.1936 | 0.318074 | -1.65256 | 0.000627 | 1.629648 |
| Com_13735_pos | Heliotrine | C16 H27 N O5 | 313.1882 | 2.658782 | 1.410766 | 0.000645 | 1.462509 |
| Com_13886_pos | cytidine 5′-monophosphate | C9 H14 N3 O8 P | 323.0505 | 0.370818 | -1.43122 | 0.000709 | 1.355073 |
| Com_2444_neg | 3-O-beta-D-Galactopyranosyl-D-arabinose | C11 H20 O10 | 312.1059 | 3.784182 | 1.919981 | 0.000735 | 1.850284 |
| Com_15809_pos | MHPG | C9 H12 O4 | 184.0737 | 3.934018 | 1.976004 | 0.000737 | 1.93601 |
| Com_12536_pos | 5-oxo-delta-bilirubin | C33 H34 N4 O7 | 598.2419 | 0.304643 | -1.71481 | 0.000755 | 1.772528 |
| Com_10766_neg | (7R)-7-(5-carboxy-5-oxopentanamido)cephalosporanic acid | C16 H18 N2 O9 S | 414.0736 | 0.283591 | -1.81812 | 0.00083 | 1.82878 |
| Com_12046_neg | 2,3,4,5-Tetrahydroxypentanal | C5 H10 O5 | 150.0528 | 4.425308 | 2.145778 | 0.000863 | 1.988558 |
| Com_1363_pos | 1-(4-Hydroxy-3-methoxyphenyl)-3,5-decanediyl diacetate | C21 H32 O6 | 380.2191 | 0.210553 | -2.24774 | 0.000914 | 2.147862 |
| Com_16139_pos | cifostodine | C9 H12 N3 O7 P | 305.0399 | 0.362993 | -1.46199 | 0.000929 | 1.375574 |
| Com_3190_pos | 17α-Hydroxyprogesterone | C21 H30 O3 | 330.2188 | 0.329995 | -1.59948 | 0.000935 | 1.620384 |
| Com_14148_neg | bezitramide | C31 H32 N4 O2 | 492.2543 | 0.263634 | -1.92339 | 0.000967 | 1.914163 |
| Com_5162_pos | Urdamycin B | C37 H44 O13 | 696.2789 | 0.233132 | -2.10078 | 0.001005 | 1.980841 |
| Com_4587_pos | Bromocriptine | C32 H40 Br N5 O5 | 653.2185 | 8.328362 | 3.058033 | 0.001076 | 2.672116 |
| Com_25368_pos | AM-Toxin I | C23 H31 N3 O6 | 445.2231 | 2.74315 | 1.455834 | 0.001113 | 1.396413 |
| Com_770_neg | Sorbitan, monododecanoate | C18 H34 O6 | 346.2358 | 3.987204 | 1.995377 | 0.001129 | 2.01833 |
| Com_9799_pos | Coumarin | C9 H6 O2 | 146.0368 | 3.506839 | 1.810171 | 0.001151 | 1.785911 |
| Com_9345_neg | 4-Hydroxy-8-(beta-D-talopyranosyloxy)-2-quinolinecarboxylic acid | C16 H17 N O9 | 367.0921 | 0.187358 | -2.41613 | 0.00122 | 2.487504 |
| Com_10629_pos | 1,5-Dihydroxy-3,4-dimethoxy-10-methyl-9(10H)-acridinone | C16 H15 N O5 | 301.0943 | 0.364379 | -1.45649 | 0.001235 | 1.433466 |
| Com_7618_neg | Geranylfarnesyl pyrophosphate | C25 H44 O7 P2 | 518.2551 | 0.369211 | -1.43748 | 0.001328 | 1.46733 |
| Com_4544_neg | Nigakilactone N | C21 H30 O7 | 394.1994 | 0.339849 | -1.55703 | 0.001361 | 1.513322 |
| Com_15014_neg | Pravastatin | C23 H36 O7 | 424.2459 | 0.452833 | -1.14295 | 0.001378 | 1.139646 |
| Com_15129_pos | Pefurazoate | C18 H23 N3 O4 | 345.1678 | 4.189945 | 2.066931 | 0.001403 | 1.89655 |
| Com_400_neg | Integerrine | C35 H39 N5 O4 | 593.3027 | 0.201887 | -2.30838 | 0.001468 | 2.555672 |
| Com_6926_neg | Clavamycin F | C15 H24 N4 O7 | 372.1657 | 2.520982 | 1.333986 | 0.001485 | 1.280217 |
| Com_8735_pos | FENAMIC ACID | C13 H11 N O2 | 213.0786 | 2.279507 | 1.188722 | 0.001518 | 1.20379 |
| Com_25924_pos | Sakuranin | C22 H24 O10 | 448.1365 | 2.477857 | 1.309093 | 0.001546 | 1.292757 |
| Com_9154_pos | dapdiamide C | C13 H22 N4 O5 | 314.1582 | 2.184008 | 1.126978 | 0.001551 | 1.105121 |
| Com_7708_neg | Gentiopicrin | C16 H20 O9 | 356.1112 | 4.636571 | 2.213058 | 0.001647 | 2.076483 |
| Com_18595_pos | benzoylagmatine | C12 H18 N4 O | 234.1476 | 2.9984 | 1.584193 | 0.001733 | 1.505936 |
| Com_9287_pos | 1-methyl-1,2,3,4-tetrahydro-beta-carboline-3-carboxylic acid | C13 H14 N2 O2 | 230.1052 | 2.379028 | 1.250372 | 0.001756 | 1.289616 |
| Com_1790_pos | 2-Amino-6-[(3-carboxypropanoyl)amino]heptanedioic acid | C11 H18 N2 O7 | 290.1101 | 6.16306 | 2.623647 | 0.001784 | 2.319778 |
| Com_5406_pos | Uroerythrin | C25 H27 N3 O6 | 465.1895 | 0.15663 | -2.67457 | 0.001833 | 2.779887 |
| Com_18849_pos | (+)-castanospermine | C8 H15 N O4 | 189.1002 | 3.376561 | 1.755555 | 0.001883 | 1.651105 |
| Com_13782_pos | Flazin | C17 H12 N2 O4 | 308.0791 | 3.063756 | 1.615301 | 0.00194 | 1.556153 |
| Com_5737_neg | Pranazepide | C26 H19 F N4 O2 | 438.1497 | 0.197308 | -2.34148 | 0.001965 | 2.196195 |
| Com_8113_neg | 3,4,5-Trihydroxy-1-cyclohexenecarboxylic acid | C7 H10 O5 | 174.0529 | 2.382395 | 1.252413 | 0.002091 | 1.218736 |
| Com_10205_neg | clocapramine | C28 H37 Cl N4 O | 480.266 | 0.350208 | -1.51372 | 0.002155 | 1.638377 |
| Com_18156_pos | APAZIQUONE | C15 H16 N2 O4 | 288.1104 | 2.282783 | 1.190794 | 0.002167 | 1.278067 |
| Com_3806_neg | Thiothixene | C23 H29 N3 O2 S2 | 443.1696 | 0.201545 | -2.31083 | 0.00217 | 2.255741 |
| Com_13730_neg | meticillin | C17 H20 N2 O6 S | 380.1045 | 0.380967 | -1.39226 | 0.002184 | 1.486251 |
| Com_1196_neg | Setipiprant | C24 H19 F N2 O3 | 402.1377 | 8.632492 | 3.109777 | 0.002191 | 2.713988 |
| Com_4001_neg | Maltopentose | C30 H52 O26 | 828.2774 | 4.485036 | 2.16512 | 0.002196 | 2.035304 |
| Com_12929_neg | barmastine | C27 H29 N7 O2 | 483.2402 | 0.222162 | -2.17032 | 0.002273 | 2.096763 |
| Com_18838_pos | porfiromycin | C16 H20 N4 O5 | 348.1424 | 2.427452 | 1.279443 | 0.00228 | 1.234848 |
| Com_3035_pos | Medroxyprogesterone 17-acetate | C24 H34 O4 | 386.2448 | 0.448939 | -1.15541 | 0.0023 | 1.148355 |
| Com_6118_neg | 7-Sulfocholic acid | C24 H40 O8 S | 488.2445 | 0.241153 | -2.05198 | 0.002316 | 2.029672 |
| Com_13179_pos | Norgestrienone | C20 H22 O2 | 294.1614 | 0.383085 | -1.38426 | 0.002349 | 1.307309 |
| Com_3066_neg | Chenodeoxycholic acid 3-sulfate | C24 H40 O7 S | 472.2496 | 0.35448 | -1.49622 | 0.002354 | 1.568248 |
| Com_1827_pos | MFCD03411993 | C18 H30 O5 | 326.2087 | 2.681758 | 1.423179 | 0.002413 | 1.474827 |
| Com_8192_pos | N~2~-(1-Carboxyethyl)-N~5~-(diaminomethylene)ornithine | C9 H18 N4 O4 | 246.1322 | 4.863157 | 2.281893 | 0.002504 | 2.131722 |
| Com_8323_pos | Tuftsin | C21 H40 N8 O6 | 500.3093 | 4.440616 | 2.15076 | 0.00256 | 2.740998 |
| Com_17093_pos | Hexyl 2-furoate | C11 H16 O3 | 196.1099 | 2.264482 | 1.179181 | 0.002562 | 1.159125 |
| Com_6701_neg | 4'-apo-beta-carotenal | C35 H46 O | 482.3541 | 0.130617 | -2.93658 | 0.00272 | 3.662354 |
| Com_21371_pos | Phoslactomycin B | C25 H40 N O8 P | 513.2507 | 0.229985 | -2.12039 | 0.002721 | 2.110541 |
| Com_21413_pos | terikalant | C24 H31 N O3 | 381.2296 | 0.434704 | -1.2019 | 0.002741 | 1.139967 |
| Com_255_pos | Palmitoylcarnitine | C23 H45 N O4 | 399.334 | 0.373651 | -1.42024 | 0.002876 | 1.477155 |
| Com_10407_pos | oxeladin | C20 H33 N O3 | 335.2453 | 2.180973 | 1.124972 | 0.002893 | 1.149905 |
| Com_5241_pos | 1-(9Z-octadecenoyl)-2-hexadecanoyl-3-beta-D-galactosyl-sn-glycerol | C43 H80 O10 | 756.5734 | 3.553821 | 1.829371 | 0.002903 | 1.814993 |
| Com_16910_pos | Glycylalanylprolylmethionylphenylalanylvalinamide | C29 H45 N7 O6 S | 619.3132 | 2.554672 | 1.353138 | 0.002906 | 1.261949 |
| Com_19310_pos | 4-(9H-beta-Carbolin-1-yl)-1,2,4-butanetriol | C15 H16 N2 O3 | 272.1157 | 3.786585 | 1.920897 | 0.002955 | 1.764552 |
| Com_6344_neg | Tenatoprazole | C16 H18 N4 O3 S | 346.1083 | 0.208331 | -2.26305 | 0.002994 | 2.07727 |
| Com_17554_pos | 7alpha-Hydroxy-3-oxochol-4-en-24-oic acid | C24 H36 O4 | 388.2603 | 0.452921 | -1.14267 | 0.003108 | 1.176758 |
| Com_10361_neg | [3-(3,4-methylenedioxyphenyl)-2-(mercaptomethyl)-1-oxoprolyl]glycine | C15 H18 N2 O6 S | 354.0889 | 0.10445 | -3.25911 | 0.003108 | 2.88669 |
| Com_21222_pos | 3-CYSTEINYLACETAMINOPHEN | C11 H14 N2 O4 S | 270.0667 | 0.383969 | -1.38094 | 0.003227 | 1.414163 |
| Com_3016_neg | Xanthommatin | C20 H13 N3 O8 | 423.0684 | 0.255732 | -1.96729 | 0.003262 | 2.200858 |
| Com_4419_neg | streptonigrin | C25 H22 N4 O8 | 506.1425 | 14.59506 | 3.867409 | 0.003342 | 3.281757 |
| Com_15275_pos | (2S)-3-Hydroxy-1,2-propanediyl dibutanoate | C11 H20 O5 | 232.1307 | 2.549979 | 1.350485 | 0.003345 | 1.241272 |
| Com_18710_pos | EUPATORIOCHROMENE | C13 H14 O3 | 218.0943 | 2.664052 | 1.413622 | 0.003375 | 1.48341 |
| Com_9197_neg | metioprim | C14 H18 N4 O2 S | 306.1139 | 3.915953 | 1.969364 | 0.003423 | 1.759362 |
| Com_7568_neg | N-formylmaleamic acid | C5 H5 N O4 | 143.022 | 2.200993 | 1.138155 | 0.003577 | 1.133895 |
| Com_18993_pos | 4,4'-(3,4-Dimethyltetrahydrofuran-2,5-diyl)bis(2-methoxyphenol) | C20 H24 O5 | 344.1615 | 0.360919 | -1.47025 | 0.003636 | 1.392939 |
| Com_5558_neg | (Z)-4-Chloro-N-(1-hydroxy-3-methoxy-3-oxopropylidene)tryptophan | C15 H15 Cl N2 O5 | 338.0659 | 2.268104 | 1.181487 | 0.00364 | 1.246705 |
| Com_7146_neg | 1-oleoyl-2-linoleoyl-sn-glycero-3-phosphocholine | C44 H82 N O8 P | 783.5751 | 4.134721 | 2.04779 | 0.003643 | 1.855702 |
| Com_21433_pos | APC | C33 H38 N4 O8 | 618.2677 | 0.394524 | -1.34181 | 0.003685 | 1.246493 |
| Com_15519_pos | 3-Methoxyestra-1,3,5(10),16-tetraene | C19 H24 O | 268.1824 | 0.459721 | -1.12117 | 0.003686 | 1.124458 |
| Com_1772_neg | Sulfazamet | C16 H16 N4 O2 S | 328.101 | 6.187894 | 2.629449 | 0.003698 | 2.261428 |
| Com_8359_neg | pinazepam | C18 H13 Cl N2 O | 308.0721 | 5.425166 | 2.439667 | 0.003736 | 2.075639 |
| Com_10665_neg | versetamide | C20 H37 N5 O10 | 507.2542 | 0.33354 | -1.58407 | 0.003795 | 1.478369 |
| Com_13599_pos | Acitretin | C21 H26 O3 | 326.1877 | 0.356173 | -1.48935 | 0.003823 | 1.43246 |
| Com_19182_pos | Aripiprazole lauroxil | C36 H51 Cl2 N3 O4 | 659.324 | 0.5118 | -0.96635 | 0.004099 | 1.003884 |
| Com_153_neg | Gluconic acid | C6 H12 O7 | 196.0584 | 16.54703 | 4.048501 | 0.004173 | 3.356916 |
| Com_10344_pos | tak-475 | C33 H41 Cl N2 O9 | 644.2523 | 0.176917 | -2.49885 | 0.004188 | 2.217127 |
| Com_8397_pos | 3-[(3-Hydroxytridecanoyl)oxy]-4-(trimethylammonio)butanoate | C20 H39 N O5 | 373.2821 | 2.954626 | 1.562975 | 0.00424 | 1.455023 |
| Com_4963_neg | (-)-secoisolariciresinol | C20 H26 O6 | 362.1732 | 0.279418 | -1.8395 | 0.004293 | 1.713461 |
| Com_312_neg | D-Raffinose | C18 H32 O16 | 504.1693 | 9.75146 | 3.285618 | 0.004414 | 2.724693 |
| Com_19474_pos | Moxisylyte | C16 H25 N O3 | 279.183 | 4.298721 | 2.103908 | 0.004582 | 1.869856 |
| Com_16880_pos | Betamipron | C10 H11 N O3 | 193.0738 | 0.426739 | -1.22857 | 0.004599 | 1.142235 |
| Com_6275_neg | cycloxazoline | C27 H42 N6 O6 | 546.3193 | 0.432744 | -1.20841 | 0.004703 | 1.135955 |
| Com_2084_neg | Aldosterone | C21 H28 O5 | 360.1939 | 0.48575 | -1.04171 | 0.004732 | 1.069402 |
| Com_3968_neg | 2,3-Bis(octylsulfanyl)-1-propanol | C19 H40 O S2 | 348.2514 | 3.242347 | 1.697039 | 0.004866 | 1.813611 |
| Com_6272_neg | isorhamnetin 3-glucoside | C22 H22 O12 | 478.1114 | 14.39659 | 3.847656 | 0.004903 | 3.24947 |
| Com_8351_neg | Baliospermin | C32 H50 O8 | 562.3487 | 0.440692 | -1.18216 | 0.004905 | 1.227695 |
| Com_623_neg | .alpha.-Glucoheptitol | C7 H16 O7 | 212.0895 | 19.48153 | 4.284035 | 0.005103 | 3.399256 |
| Com_3636_neg | Asebotoxin II | C23 H36 O6 | 408.2514 | 0.284479 | -1.81361 | 0.005129 | 2.004969 |
| Com_3661_neg | Deacetoxy(7)-7-Oxokhivorinic Acid | C27 H36 O10 | 520.2277 | 0.279036 | -1.84148 | 0.005169 | 1.682183 |
| Com_1588_pos | Dibenzothiophene | C12 H8 S | 184.0346 | 4.954417 | 2.308715 | 0.005287 | 2.092582 |
| Com_6231_pos | DIGITOGENIN | C27 H44 O5 | 448.3184 | 0.391749 | -1.352 | 0.005384 | 1.240502 |
| Com_3791_neg | Elesclomol | C19 H20 N4 O2 S2 | 400.1027 | 13.1268 | 3.714443 | 0.005529 | 3.116402 |
| Com_7097_neg | Nebramycin IV | C19 H38 N6 O11 | 526.2598 | 0.415889 | -1.26573 | 0.005588 | 1.196718 |
| Com_19035_pos | NL8513000 | C11 H13 N O2 | 191.0946 | 3.645078 | 1.86595 | 0.005596 | 1.695863 |
| Com_529_neg | 4-O-(beta-L-Araf)-cis-L-Hyp | C10 H17 N O7 | 263.1006 | 0.465445 | -1.10332 | 0.005809 | 1.044371 |
| Com_322_pos | N-Acetylneuraminic acid | C11 H19 N O9 | 309.1051 | 0.462294 | -1.11312 | 0.005836 | 1.096372 |
| Com_638_pos | D-(+)-Maltose | C12 H22 O11 | 364.0971 | 5.631284 | 2.493464 | 0.006045 | 2.220607 |
| Com_248_pos | MFCD22416941 | C25 H47 N O4 | 425.3497 | 0.346581 | -1.52874 | 0.006256 | 1.576788 |
| Com_2674_pos | N-α-L-Acetyl-arginine | C8 H16 N4 O3 | 216.1222 | 3.065689 | 1.616212 | 0.006323 | 1.414439 |
| Com_3114_neg | coformycin | C11 H16 N4 O5 | 284.1109 | 2.517969 | 1.332261 | 0.006324 | 1.189655 |
| Com_19482_pos | 5''-phosphoribostamycin | C17 H35 N4 O13 P | 534.1957 | 2.44448 | 1.289527 | 0.006362 | 1.34622 |
| Com_10278_neg | Zaleplon | C17 H15 N5 O | 305.1265 | 0.473865 | -1.07745 | 0.006436 | 1.151894 |
| Com_6372_neg | candidone | C22 H24 O4 | 352.1676 | 7.074868 | 2.822703 | 0.006449 | 2.408888 |
| Com_1864_pos | stearoylcarnitine | C25 H49 N O4 | 427.3653 | 0.398913 | -1.32585 | 0.006579 | 1.279476 |
| Com_5542_neg | Hydrocortisone Valerate | C26 H38 O6 | 446.2671 | 0.368966 | -1.43844 | 0.0066 | 1.31662 |
| Com_13307_pos | Protoverine | C27 H43 N O9 | 525.2941 | 0.215556 | -2.21387 | 0.006924 | 1.941264 |
| Com_4938_neg | ARAMITE | C15 H23 Cl O4 S | 334.1014 | 3.651008 | 1.868295 | 0.006992 | 1.787689 |
| Com_5707_neg | Urolithin B 3-O-glucuronide | C19 H16 O9 | 388.0789 | 4.688021 | 2.228979 | 0.007143 | 2.117661 |
| Com_7191_pos | 4-amino-2-hydroxyamino-6-nitrotoluene | C7 H9 N3 O3 | 183.0642 | 2.364897 | 1.241777 | 0.007224 | 1.162388 |
| Com_19997_pos | Rhodoxanthin | C40 H50 O2 | 562.3821 | 2.895347 | 1.533736 | 0.007235 | 1.364337 |
| Com_1792_neg | Tyrosol | C8 H10 O2 | 138.0683 | 0.022247 | -5.49025 | 0.007235 | 4.445102 |
| Com_28810_pos | Pentosidine | C17 H26 N6 O4 | 378.2033 | 0.403418 | -1.30965 | 0.007329 | 1.242066 |
| Com_3487_neg | Gemfibrozil | C15 H22 O3 | 250.157 | 3.570633 | 1.83618 | 0.007396 | 1.706931 |
| Com_530_neg | Lithocholic acid taurine conjugate | C26 H45 N O5 S | 483.302 | 4.465827 | 2.158927 | 0.007423 | 2.24584 |
| Com_7288_pos | Tapentadol | C14 H23 N O | 221.1775 | 0.414911 | -1.26913 | 0.007586 | 1.137755 |
| Com_9563_neg | Kanokoside D | C27 H44 O16 | 624.2617 | 0.366905 | -1.44652 | 0.00759 | 1.28853 |
| Com_2808_neg | prostaglandin G2 2-glyceryl ester | C23 H38 O8 | 442.2568 | 0.183631 | -2.44512 | 0.007591 | 2.069085 |
| Com_6752_neg | 2-methoxy-6-all-trans-heptaprenylhydroquinonel | C42 H64 O3 | 616.4832 | 2.329331 | 1.219916 | 0.00761 | 1.188676 |
| Com_2291_neg | proquinolate | C17 H21 N O5 | 319.1421 | 0.364673 | -1.45532 | 0.007735 | 1.542609 |
| Com_1909_pos | curine | C36 H38 N2 O6 | 594.2739 | 0.193749 | -2.36774 | 0.007785 | 2.240073 |
| Com_3022_neg | 1-stearyl estercitric acid | C24 H44 O7 | 444.3089 | 0.233777 | -2.09679 | 0.007808 | 1.863877 |
| Com_3291_neg | fructosylglycine | C8 H15 N O7 | 237.085 | 3.720232 | 1.895392 | 0.007861 | 1.62792 |
| Com_9283_pos | chi,chi-Caroten-18-ol | C40 H48 O | 544.3722 | 3.036705 | 1.602507 | 0.007996 | 1.524897 |
| Com_3335_pos | Furaneol | C6 H8 O3 | 128.0474 | 2.862093 | 1.51707 | 0.008034 | 1.742862 |
| Com_12446_neg | Tiotidine | C10 H16 N8 S2 | 312.0951 | 0.262198 | -1.93127 | 0.008146 | 1.72052 |
| Com_783_pos | Glycitein | C16 H12 O5 | 284.0679 | 4.043055 | 2.015446 | 0.008275 | 2.356746 |
| Com_321_pos | malonylgenistin | C24 H22 O13 | 518.1056 | 133.9559 | 7.065614 | 0.008416 | 5.654222 |
| Com_17088_pos | Eslicarbazepine | C15 H14 N2 O2 | 254.1051 | 0.415229 | -1.26802 | 0.008594 | 1.161085 |
| Com_5322_neg | Nepaline | C36 H51 N O12 | 689.3406 | 0.181556 | -2.46151 | 0.008662 | 2.63966 |
| Com_2165_neg | ASCORBIC ACID | C6 H8 O6 | 176.0322 | 0.266436 | -1.90814 | 0.009067 | 2.033951 |
| Com_12852_pos | Balfourodinine | C17 H22 N O4 | 304.1529 | 4.53219 | 2.180208 | 0.00907 | 2.336173 |
| Com_8758_neg | tectoridin | C22 H22 O11 | 462.1165 | 10.089 | 3.334712 | 0.0091 | 2.721854 |
| Com_8021_pos | Ethanoic anhydride | C4 H6 O3 | 102.0319 | 2.205758 | 1.141274 | 0.009231 | 1.037603 |
| Com_6060_pos | 1,3-Diphenylpropyl hydrogen sulfate | C15 H16 O4 S | 292.0761 | 4.011601 | 2.004178 | 0.009317 | 1.703244 |
| Com_1324_pos | Biliverdin | C33 H34 N4 O6 | 582.2469 | 0.128638 | -2.95861 | 0.00934 | 2.507401 |
| Com_145_neg | D-(-)-Ribose | C5 H10 O5 | 150.053 | 4.458722 | 2.15663 | 0.009531 | 1.826068 |
| Com_2892_neg | nostocyclamide | C20 H22 N6 O4 S2 | 474.1161 | 9.054874 | 3.178695 | 0.009551 | 2.670948 |
| Com_462_pos | (R)-Equol | C15 H14 O3 | 242.0939 | 7.513678 | 2.909519 | 0.009635 | 2.482371 |
| Com_1226_pos | N-(3-Carboxypropanoyl)-5-hydroxynorvaline | C9 H15 N O6 | 233.0893 | 5.792547 | 2.534198 | 0.009837 | 2.189381 |
| Com_4994_neg | 4-(2-Hydroxyethyl)phenyl hydrogen sulfate | C8 H10 O5 S | 218.025 | 0.082669 | -3.5965 | 0.009981 | 2.935219 |
| Com_24328_pos | 3-(6-Amino-9H-purin-9-yl)-2-nonanol | C14 H23 N5 O | 277.1891 | 0.210218 | -2.25004 | 0.01 | 1.86538 |
| Com_2998_neg | AMCC | C7 H12 N2 O4 S | 220.0528 | 5.272039 | 2.398361 | 0.01011 | 2.643476 |
| Com_3039_pos | 4-(2-Carboxy-1-azetidinyl)-N-(3-carboxy-3-hydroxypropyl)threonine | C12 H20 N2 O8 | 320.121 | 4.110584 | 2.039344 | 0.010198 | 1.685612 |
| Com_3155_neg | Secogalioside | C17 H24 O12 | 420.1263 | 0.225563 | -2.1484 | 0.010266 | 2.190408 |
| Com_351_neg | Kukoamine A | C28 H42 N4 O6 | 530.31 | 0.445115 | -1.16775 | 0.010446 | 1.349177 |
| Com_12931_neg | S-(PGA1)-glutathione | C30 H49 N3 O10 S | 643.3111 | 0.305619 | -1.71019 | 0.010453 | 1.74823 |
| Com_14262_neg | 1,2-Dimethoxy-13-methyl[1,3]benzodioxolo[5,6-c]phenanthridine | C21 H17 N O4 | 347.1156 | 0.346978 | -1.52708 | 0.010466 | 1.314092 |
| Com_6620_pos | 1,2,3,4-tetrahydro-beta-carboline-3-carboxylic acid | C12 H12 N2 O2 | 216.0897 | 4.388222 | 2.133637 | 0.010579 | 2.070413 |
| Com_2734_pos | 1-Methyladenosine | C11 H15 N5 O4 | 281.1105 | 2.435749 | 1.284366 | 0.010589 | 1.179034 |
| Com_4128_neg | Hexanoic acid | C6 H12 O2 | 116.0839 | 0.459011 | -1.1234 | 0.010693 | 1.247502 |
| Com_19857_pos | Zolmitriptan | C16 H21 N3 O2 | 287.1626 | 2.255154 | 1.173226 | 0.010738 | 1.177805 |
| Com_15830_pos | N-Acetyl-L-cysteine | C5 H9 N O3 S | 163.0303 | 0.367895 | -1.44263 | 0.010739 | 1.264587 |
| Com_15061_neg | SB-228357 | C22 H17 F4 N3 O2 | 431.1252 | 0.470848 | -1.08667 | 0.010773 | 1.107894 |
| Com_763_pos | Kynurenic acid | C10 H7 N O3 | 189.0425 | 3.909329 | 1.966921 | 0.010972 | 1.881718 |
| Com_4138_neg | 4-O-beta-D-Xylopyranosyl-D-xylopyranose | C10 H18 O9 | 282.0951 | 3.596562 | 1.846619 | 0.011009 | 1.601604 |
| Com_7751_neg | 1-palmitoylglycerone 3-phosphate | C19 H37 O7 P | 408.2279 | 0.270271 | -1.88752 | 0.011089 | 1.742266 |
| Com_2623_neg | Diosgenin | C27 H42 O3 | 414.3136 | 0.359649 | -1.47534 | 0.011107 | 1.638665 |
| Com_5576_pos | R41207 | C13 H10 O3 | 214.0622 | 2.264707 | 1.179324 | 0.011187 | 1.041518 |
| Com_15229_pos | ALLN | C20 H37 N3 O4 | 383.2776 | 2.016083 | 1.011555 | 0.011308 | 1.108759 |
| Com_4552_pos | 13-Hydroxykaur-16-en-18-oic acid | C20 H30 O3 | 318.2188 | 0.396001 | -1.33642 | 0.011608 | 1.196268 |
| Com_7095_pos | (E)-2-[(2S)-2-Amino-2-carboxyethoxy]-2-hydroxyethenediazonium | C5 H8 N3 O4 | 174.0502 | 4.710983 | 2.236028 | 0.011642 | 2.050647 |
| Com_9724_pos | Panthenyl ethyl ether | C11 H23 N O4 | 233.1624 | 2.014087 | 1.010126 | 0.011776 | 1.180351 |
| Com_22724_pos | Cammaconine | C23 H37 N O5 | 407.2662 | 0.198795 | -2.33065 | 0.011865 | 1.863051 |
| Com_9632_pos | (E)-4-Methoxycinnamic acid | C10 H10 O3 | 178.0629 | 2.314989 | 1.211005 | 0.011871 | 1.093766 |
| Com_24387_pos | evatanepag | C25 H28 N2 O5 S | 468.1739 | 3.185074 | 1.671327 | 0.011994 | 1.486317 |
| Com_12885_neg | GSK4716 | C17 H18 N2 O2 | 282.1377 | 0.456311 | -1.13191 | 0.012098 | 1.149612 |
| Com_1687_neg | 1-O-acetyl-alpha-maltose | C14 H24 O12 | 384.1271 | 3.771 | 1.914947 | 0.012371 | 1.621003 |
| Com_21113_pos | (Z)-3-butylidenephthalide | C12 H12 O2 | 188.0836 | 2.494357 | 1.318668 | 0.012476 | 1.178959 |
| Com_27695_pos | estrofurate | C24 H26 O4 | 378.1817 | 4.893641 | 2.290908 | 0.012516 | 1.837071 |
| Com_3087_pos | Choline O-Sulfate | C5 H13 N O4 S | 183.0564 | 6.361569 | 2.669383 | 0.012586 | 2.334327 |
| Com_6098_neg | Nisterime acetate | C27 H35 Cl N2 O5 | 502.2237 | 0.437948 | -1.19117 | 0.012813 | 1.29926 |
| Com_21911_pos | Arachidonyl trifluoromethyl ketone | C21 H31 F3 O | 356.2317 | 0.314269 | -1.66993 | 0.012941 | 1.496799 |
| Com_4245_neg | Leukotriene E3 | C23 H39 N O5 S | 441.2551 | 0.260919 | -1.93832 | 0.013008 | 1.669986 |
| Com_1081_neg | Prostaglandin F2α 1-11-lactone | C20 H32 O4 | 336.2303 | 0.367329 | -1.44485 | 0.01302 | 1.536292 |
| Com_4209_neg | 2,6-Dideoxy-3-O-methyl-L-arabino-hexopyranose | C7 H14 O4 | 162.0893 | 4.92539 | 2.300238 | 0.013236 | 1.92385 |
| Com_6103_pos | Dolichyl b-D-glucosyl phosphate | C21 H39 O9 P | 466.2322 | 0.31178 | -1.6814 | 0.013248 | 1.664744 |
| Com_10118_neg | UDP-GlcNAc | C17 H27 N3 O17 P2 | 607.0827 | 0.248821 | -2.00682 | 0.013269 | 1.747183 |
| Com_8507_pos | Crizotinib | C21 H22 Cl2 F N5 O | 449.1195 | 3.803662 | 1.927389 | 0.013581 | 1.566555 |
| Com_266_pos | Linoleyl carnitine | C25 H45 N O4 | 423.334 | 0.191657 | -2.3834 | 0.013585 | 2.076257 |
| Com_4999_neg | Deltorphin 1 | C37 H52 N8 O10 | 768.3831 | 0.346593 | -1.52868 | 0.013609 | 1.492441 |
| Com_8163_neg | 9-fluoro-17-methyl-5beta-androstane-3alpha,11beta,17beta-triol | C20 H33 F O3 | 340.2425 | 2.286822 | 1.193344 | 0.013679 | 1.206861 |
| Com_1368_neg | marrubin | C20 H28 O4 | 332.1989 | 0.101023 | -3.30724 | 0.013827 | 2.432207 |
| Com_13541_pos | Cuauhtemone | C15 H24 O3 | 252.172 | 2.657584 | 1.410115 | 0.013874 | 1.296016 |
| Com_2975_neg | Lipoamide | C8 H15 N O S2 | 205.0587 | 2.710013 | 1.4383 | 0.01388 | 1.348628 |
| Com_12902_pos | Diacetin | C7 H12 O5 | 176.0684 | 2.050685 | 1.036106 | 0.013882 | 1.124009 |
| Com_14762_neg | 3-O-(alpha-L-oleandrosyl)oleandolide | C27 H46 O10 | 530.3067 | 0.469306 | -1.0914 | 0.013886 | 1.069576 |
| Com_25_neg | Retinyl acetate | C22 H32 O2 | 328.2403 | 0.466175 | -1.10106 | 0.014067 | 1.134733 |
| Com_4523_pos | Androsterone | C19 H30 O2 | 290.2241 | 3.967423 | 1.988202 | 0.014169 | 1.69967 |
| Com_10627_neg | fluvastatin | C24 H26 F N O4 | 411.1832 | 0.22941 | -2.124 | 0.014335 | 2.107891 |
| Com_1971_pos | Quinaprilat | C23 H26 N2 O5 | 410.1834 | 9.645735 | 3.269891 | 0.014614 | 2.959001 |
| Com_441_neg | Sucrose | C12 H22 O11 | 342.1165 | 3.280561 | 1.713943 | 0.014626 | 1.483563 |
| Com_432_neg | Quebrachitol | C7 H14 O6 | 194.0792 | 3.4687 | 1.794395 | 0.014721 | 1.697964 |
| Com_1688_neg | Barbinine | C36 H46 N2 O10 | 666.3153 | 0.282933 | -1.82147 | 0.014904 | 2.242185 |
| Com_1694_pos | Corticosterone | C21 H30 O4 | 346.2136 | 0.205376 | -2.28366 | 0.01498 | 1.986981 |
| Com_3383_neg | Gedocarnil | C23 H21 Cl N2 O4 | 424.1196 | 2.880258 | 1.526198 | 0.015109 | 1.338356 |
| Com_1103_pos | N-acetyl(phenyl)cysteine | C11 H13 N O3 S | 239.0611 | 0.01335 | -6.22702 | 0.015114 | 4.69036 |
| Com_315_pos | Daidzein | C15 H10 O4 | 254.0574 | 4.187492 | 2.066086 | 0.015283 | 2.362567 |
| Com_4039_neg | Rehmaionoside C | C19 H32 O8 | 388.21 | 4.466896 | 2.159273 | 0.015548 | 1.709061 |
| Com_9399_neg | deaminohydroxyblasticidin S | C17 H25 N7 O6 | 423.1855 | 0.447251 | -1.16084 | 0.015659 | 1.08237 |
| Com_27066_pos | pirenperone | C23 H24 F N3 O2 | 393.1862 | 0.373959 | -1.41905 | 0.015799 | 1.724149 |
| Com_70_pos | 1-Linoleoyl-sn-glycero-3-phosphocholine | C26 H50 N O7 P | 519.332 | 2.064164 | 1.045558 | 0.015831 | 1.180757 |
| Com_1551_neg | 3-(acetamidomethylidene)-2-(hydroxymethyl)succinic acid | C8 H11 N O6 | 217.0586 | 4.21395 | 2.075173 | 0.015882 | 1.979367 |
| Com_22285_pos | L-Theanine | C7 H14 N2 O3 | 174.1004 | 2.384537 | 1.253709 | 0.015885 | 1.14281 |
| Com_13712_neg | Batrachotoxin | C31 H42 N2 O6 | 538.3058 | 0.339899 | -1.55682 | 0.016061 | 1.821066 |
| Com_1715_neg | 6-Hydroxy-3-methyl-9-(beta-D-ribofuranosyl)-3,9-dihydro-2H-purin-2-one | C11 H14 N4 O6 | 298.0902 | 4.413175 | 2.141817 | 0.016285 | 1.866354 |
| Com_251_neg | Equol | C15 H14 O3 | 242.0945 | 7.510156 | 2.908843 | 0.016319 | 2.324845 |
| Com_19437_pos | 1-Myristoyl-sn-glycerol 3-phosphate | C17 H35 O7 P | 382.2115 | 0.312414 | -1.67847 | 0.016349 | 1.394978 |
| Com_4212_pos | β-Asarone | C12 H16 O3 | 208.1099 | 4.471918 | 2.160894 | 0.016397 | 1.943911 |
| Com_619_neg | N-Glycolylneuraminic acid | C11 H19 N O10 | 325.101 | 0.409005 | -1.28981 | 0.016429 | 1.390217 |
| Com_1571_pos | Malonylglycitin | C25 H24 O13 | 532.1214 | 31.34375 | 4.970106 | 0.016454 | 3.570257 |
| Com_19220_pos | 2-Phenyl-4-pentenal | C11 H12 O | 160.0887 | 2.631576 | 1.395927 | 0.016593 | 1.226485 |
| Com_4873_pos | Succinyl proline | C9 H13 N O5 | 215.0789 | 0.369433 | -1.43661 | 0.016627 | 1.389603 |
| Com_388_neg | 1-stearoyl-sn-glycero-3-phospho-1D-myo-inositol | C27 H53 O12 P | 600.3278 | 0.436347 | -1.19645 | 0.01704 | 1.458096 |
| Com_2328_pos | Hexitol | C6 H14 O6 | 182.0789 | 11.16677 | 3.48114 | 0.017148 | 2.692559 |
| Com_547_pos | malonyldaidzin | C24 H22 O12 | 502.1107 | 155.5624 | 7.281349 | 0.017232 | 5.164673 |
| Com_721_neg | 1-Naphthylacetylspermine | C22 H34 N4 O | 370.2722 | 3.624436 | 1.857756 | 0.017364 | 1.807187 |
| Com_126_neg | D-(-)-Mannitol | C6 H14 O6 | 182.0793 | 6.386721 | 2.675075 | 0.017365 | 3.275244 |
| Com_1510_neg | Maltitol | C12 H24 O11 | 344.1321 | 4.219638 | 2.077119 | 0.017501 | 1.793211 |
| Com_5698_pos | Hexadecanedioic acid mono-L-carnitine ester | C23 H43 N O6 | 429.3083 | 2.004215 | 1.003037 | 0.017613 | 1.037555 |
| Com_17537_pos | Ulodesine | C12 H16 N4 O3 | 264.1215 | 3.994649 | 1.998069 | 0.017633 | 1.739619 |
| Com_7487_pos | Dihexyverine | C20 H35 N O2 | 321.2661 | 2.702779 | 1.434443 | 0.017635 | 1.580708 |
| Com_8489_pos | Misonidazole | C7 H11 N3 O4 | 201.0747 | 2.012647 | 1.009094 | 0.017755 | 1.038235 |
| Com_827_pos | Docosatetraenoic acid | C22 H36 O2 | 332.2708 | 0.14726 | -2.76356 | 0.018043 | 3.368109 |
| Com_172_neg | MFCD00009868 | C16 H28 O2 | 252.2089 | 0.444321 | -1.17033 | 0.018112 | 1.177934 |
| Com_1744_neg | IY9RJD7JGI | C18 H20 Cl2 N2 O3 | 382.0839 | 0.27135 | -1.88177 | 0.018211 | 1.672175 |
| Com_12310_pos | Fingolimod | C19 H33 N O2 | 307.2505 | 2.582442 | 1.368736 | 0.018227 | 1.195177 |
| Com_8984_pos | Urdamycin G | C37 H46 O14 | 714.2879 | 0.330992 | -1.59513 | 0.01842 | 1.401848 |
| Com_12190_pos | 4-(3,5-Diphenylcyclohexyl)phenol | C24 H24 O | 328.1817 | 2.808123 | 1.489606 | 0.018436 | 1.352019 |
| Com_7419_neg | 2-Oxo-3-(phosphonooxy)propyl decanoate | C13 H25 O7 P | 324.1325 | 3.532995 | 1.820892 | 0.018487 | 1.826272 |
| Com_2685_pos | pratosartan | C25 H26 N6 O | 426.2179 | 0.179932 | -2.47448 | 0.01852 | 2.205794 |
| Com_4464_pos | 5-Hydroxyindole | C8 H7 N O | 133.0528 | 2.527257 | 1.337572 | 0.01869 | 1.215717 |
| Com_4293_neg | Sulforhodamine B | C27 H30 N2 O7 S2 | 558.1496 | 3.587225 | 1.842868 | 0.018973 | 1.76824 |
| Com_5325_pos | Saracatinib | C27 H32 Cl N5 O5 | 541.2097 | 0.250908 | -1.99477 | 0.019022 | 1.730881 |
| Com_3822_pos | muramic acid | C9 H17 N O7 | 251.0998 | 4.622634 | 2.208715 | 0.019046 | 1.805134 |
| Com_7595_pos | Tisocalcitate | C31 H48 O5 | 500.3493 | 0.397794 | -1.32991 | 0.019125 | 1.181137 |
| Com_5189_neg | p-Toluenesulfonic acid | C7 H8 O3 S | 172.0195 | 3.232197 | 1.692515 | 0.019431 | 1.475991 |
| Com_2051_pos | Androstanolone | C19 H30 O2 | 290.224 | 3.014688 | 1.592009 | 0.019605 | 1.560197 |
| Com_671_neg | Diethylpyrocarbonate | C6 H10 O5 | 162.0531 | 2.391283 | 1.257785 | 0.019646 | 1.314214 |
| Com_4931_pos | Benzaldehyde | C7 H6 O | 106.0421 | 3.433799 | 1.779805 | 0.019747 | 1.445803 |
| Com_972_neg | 5-HIAA | C10 H9 N O3 | 191.0585 | 4.427337 | 2.146439 | 0.019897 | 2.120789 |
| Com_1850_neg | 1-tetradecyl-2-[(9Z)-hexadecenoyl]-sn-glycero-3-phosphocholine | C38 H76 N O7 P | 689.5332 | 2.418486 | 1.274104 | 0.020131 | 1.160746 |
| Com_1894_neg | Calcitriol | C27 H44 O3 | 416.3292 | 0.342377 | -1.54634 | 0.020388 | 1.662298 |
| Com_585_pos | 3-hydroxyhexadecanoylcarnitine | C23 H45 N O5 | 415.3289 | 0.215092 | -2.21697 | 0.020544 | 2.003616 |
| Com_9950_neg | devapamil | C26 H36 N2 O3 | 424.2739 | 0.304734 | -1.71438 | 0.0208 | 1.698715 |
| Com_29427_pos | Sophoramine | C15 H20 N2 O | 244.1573 | 3.094423 | 1.62967 | 0.020815 | 1.384204 |
| Com_15894_pos | tiacrilast | C12 H10 N2 O3 S | 262.0408 | 0.13814 | -2.85579 | 0.020903 | 2.169327 |
| Com_6866_pos | ESTRADIOL PHENYLPROPIONATE | C27 H32 O3 | 404.2337 | 0.156682 | -2.67409 | 0.02097 | 2.223596 |
| Com_9360_neg | doxacurium | C56 H78 N2 O16 | 1034.535 | 0.303565 | -1.71992 | 0.021079 | 1.44619 |
| Com_9499_neg | Lisinopril | C21 H31 N3 O5 | 405.2267 | 3.773558 | 1.915925 | 0.021087 | 1.716508 |
| Com_8514_pos | Glyceryl 2-myristate | C17 H34 O4 | 302.2448 | 0.394429 | -1.34216 | 0.021247 | 1.183904 |
| Com_15434_pos | (2R,3S)-3-Hydroxy-8-methyl-8-azabicyclo[3.2.1]octane-2-carboxylic acid | C9 H15 N O3 | 185.1051 | 2.178044 | 1.123033 | 0.021327 | 1.027015 |
| Com_18493_pos | Bergenin | C14 H16 O9 | 328.0789 | 0.385085 | -1.37675 | 0.021372 | 1.181365 |
| Com_1958_pos | folinic acid | C20 H23 N7 O7 | 473.1651 | 6.877847 | 2.781957 | 0.021425 | 2.922827 |
| Com_2376_pos | Butopyronoxyl | C12 H18 O4 | 226.1202 | 3.926427 | 1.973217 | 0.02171 | 1.82231 |
| Com_4207_pos | Bicine | C6 H13 N O4 | 163.0844 | 2.467067 | 1.302797 | 0.021882 | 1.289372 |
| Com_3724_neg | (3E)-3-Methyl-2-oxo-4-phenyl-3-buten-1-yl hydrogen sulfate | C11 H12 O5 S | 256.0406 | 2.294623 | 1.198257 | 0.022067 | 1.328847 |
| Com_5213_neg | Pyrenophorol | C16 H24 O6 | 312.1521 | 0.252684 | -1.98459 | 0.022074 | 1.681205 |
| Com_1752_neg | Lactobionic acid | C12 H22 O12 | 358.1113 | 4.743506 | 2.245954 | 0.02208 | 1.957536 |
| Com_11088_pos | Prunin | C21 H22 O10 | 434.1203 | 3.363051 | 1.74977 | 0.022261 | 1.939132 |
| Com_1683_neg | 9-[(methylthio)nonyl]thiohydroximate | C10 H21 N O S2 | 235.1057 | 0.434058 | -1.20404 | 0.022673 | 1.266487 |
| Com_273_pos | D-Sphingosine | C18 H37 N O2 | 299.2818 | 0.332742 | -1.58752 | 0.023146 | 1.504848 |
| Com_19913_pos | Suxibuzone | C24 H26 N2 O6 | 438.1777 | 2.38323 | 1.252918 | 0.023532 | 1.11572 |
| Com_19699_pos | Meclizine | C25 H27 Cl N2 | 390.1857 | 0.211767 | -2.23945 | 0.02361 | 2.157257 |
| Com_1298_neg | 6-O-Sulfo-alpha-D-galactopyranose | C6 H12 O9 S | 260.0202 | 0.28295 | -1.82138 | 0.023848 | 1.766449 |
| Com_3420_neg | bolmantalate | C29 H40 O3 | 436.2957 | 0.296835 | -1.75227 | 0.024256 | 2.031557 |
| Com_1066_neg | Jujuboside B | C52 H84 O21 | 1044.55 | 4.069242 | 2.02476 | 0.024449 | 2.206433 |
| Com_4750_neg | streptothricin F acid | C19 H36 N8 O9 | 520.2593 | 2.862282 | 1.517166 | 0.02464 | 1.62723 |
| Com_8618_pos | Lacto-N-tetraose | C26 H45 N O21 | 707.2463 | 0.456468 | -1.13141 | 0.024774 | 1.045872 |
| Com_16583_pos | Isorenieratene | C40 H48 | 528.3779 | 2.386192 | 1.25471 | 0.025149 | 1.117718 |
| Com_5838_pos | Azidopine | C27 H26 F3 N5 O5 | 557.1885 | 6.08605 | 2.605506 | 0.025324 | 2.696539 |
| Com_12338_pos | Methyl (3-hydroxy-2-oxo-2,3-dihydro-1H-indol-3-yl)acetate | C11 H11 N O4 | 221.0686 | 2.64112 | 1.40115 | 0.025745 | 1.592122 |
| Com_1889_neg | 1,3,4,5-Tetrahydroxycyclohexanecarboxylic acid | C7 H12 O6 | 192.0636 | 2.8048 | 1.487898 | 0.025758 | 1.251576 |
| Com_156_pos | L-Tyrosine | C9 H11 N O3 | 181.0738 | 1.902321 | 0.927761 | 0.025797 | 1.012804 |
| Com_395_pos | Coumarone | C8 H6 O | 118.0419 | 1.897369 | 0.924 | 0.025928 | 1.037945 |
| Com_14074_pos | 2444643WXK | C13 H18 O4 | 238.1201 | 2.672777 | 1.41834 | 0.026136 | 1.157024 |
| Com_26757_pos | 2-(tert-butylamino)-1-(3-chlorophenyl)propan-1-ol | C13 H20 Cl N O | 241.1242 | 2.15618 | 1.108478 | 0.026222 | 1.026729 |
| Com_2370_neg | 4-carboxy-4'-sulfoazobenzene | C13 H10 N2 O5 S | 306.0304 | 0.273262 | -1.87165 | 0.026284 | 1.833218 |
| Com_10784_pos | Tetralin | C10 H12 | 132.0939 | 2.111347 | 1.078164 | 0.026592 | 1.16568 |
| Com_5405_pos | Xanthurenic acid | C10 H7 N O4 | 205.0373 | 2.297045 | 1.199779 | 0.02674 | 1.275901 |
| Com_350_pos | (2E)-2,5-Dichloro-4-oxo-2-hexenedioic acid | C6 H4 Cl2 O5 | 225.9438 | 0.440975 | -1.18123 | 0.026988 | 1.174816 |
| Com_1054_neg | tetradec-5-ynoic acid | C14 H24 O2 | 224.1779 | 0.376383 | -1.40973 | 0.026996 | 1.419804 |
| Com_1162_pos | Asarone | C12 H16 O3 | 208.1097 | 2.39633 | 1.260827 | 0.026997 | 1.438997 |
| Com_7308_neg | 1-arachidonoyl-sn-glycerol 3-phosphate | C23 H39 O7 P | 458.2452 | 0.430925 | -1.21449 | 0.027133 | 1.178058 |
| Com_4021_neg | Digitoxin | C41 H64 O13 | 764.4375 | 3.321804 | 1.731967 | 0.027314 | 1.655191 |
| Com_27809_pos | Cloprostenol | C22 H29 Cl O6 | 424.1665 | 2.189315 | 1.130479 | 0.027409 | 1.006077 |
| Com_7803_neg | (Z)-4-Chloro-N-(3-ethoxy-1-hydroxy-3-oxopropylidene)tryptophan | C16 H17 Cl N2 O5 | 352.0817 | 2.213836 | 1.146549 | 0.027516 | 1.248791 |
| Com_4628_pos | Decylubiquinone | C19 H30 O4 | 322.2136 | 3.285578 | 1.716147 | 0.027728 | 1.439887 |
| Com_12130_neg | SULBACTAM PIVOXIL | C14 H21 N O7 S | 347.1039 | 0.421896 | -1.24504 | 0.027908 | 1.166602 |
| Com_317_pos | 5-Hydroxyindole-3-acetic acid | C10 H9 N O3 | 191.0581 | 4.268716 | 2.093802 | 0.028047 | 2.320856 |
| Com_5009_pos | 3β-Androstanediol | C19 H32 O2 | 292.2397 | 0.057733 | -4.11446 | 0.028142 | 2.949655 |
| Com_1291_neg | 3-Hydroxy-2-methyl-2-[(sulfooxy)methyl]propanoic acid | C5 H10 O7 S | 214.0147 | 0.299991 | -1.73701 | 0.028492 | 1.676115 |
| Com_16552_pos | L-pyrrolysine | C12 H21 N3 O3 | 255.1578 | 0.270944 | -1.88393 | 0.028589 | 1.539994 |
| Com_2334_pos | ONONETIN | C15 H14 O4 | 258.0888 | 6.805487 | 2.766698 | 0.028629 | 2.278704 |
| Com_723_pos | BIS-FERULAMIDOBUTANE | C24 H28 N2 O6 | 440.1941 | 7.075146 | 2.82276 | 0.02867 | 2.729637 |
| Com_14275_neg | Desoxymycin | C21 H41 N7 O11 | 567.2871 | 0.370161 | -1.43378 | 0.028737 | 1.296189 |
| Com_18711_pos | 2'-oxokanamycin | C18 H34 N4 O11 | 482.2238 | 0.337919 | -1.56525 | 0.028913 | 1.389994 |
| Com_11287_pos | 3-Phenylpropionitrile | C9 H9 N | 131.0736 | 0.170284 | -2.55398 | 0.029074 | 1.986862 |
| Com_7479_pos | (3S)-3-hydroxy-L-enduracididine | C6 H12 N4 O3 | 188.0913 | 3.912671 | 1.968154 | 0.029114 | 1.588588 |
| Com_508_neg | 4-Toluic acid | C8 H8 O2 | 136.0527 | 0.035 | -4.83652 | 0.029147 | 3.349428 |
| Com_10699_pos | 2-methoxyacetaminophen sulfate | C9 H11 N O6 S | 261.0306 | 3.009576 | 1.58956 | 0.029531 | 1.406342 |
| Com_8552_pos | 5-Formyl-2-furoic acid | C6 H4 O4 | 140.0109 | 0.383699 | -1.38195 | 0.029619 | 1.330842 |
| Com_11077_neg | Vaspit | C26 H35 F O5 | 446.2452 | 0.32428 | -1.62469 | 0.029921 | 1.40891 |
| Com_1661_neg | Acetylcarnitine | C9 H17 N O4 | 203.116 | 7.491175 | 2.905192 | 0.030338 | 2.155005 |
| Com_14094_pos | (2R,3S,3'S)-2,3,3'-Trihydroxy-beta,beta-carotene-4,4'-dione | C40 H52 O5 | 612.3828 | 4.576491 | 2.194242 | 0.030369 | 1.928774 |
| Com_6676_neg | MFCD00273304 | C21 H30 O6 | 378.2043 | 0.52919 | -0.91814 | 0.030453 | 1.045189 |
| Com_3492_neg | Safrole | C10 H10 O2 | 162.0682 | 0.093198 | -3.42355 | 0.030463 | 2.500775 |
| Com_3391_neg | Bufotalin | C26 H36 O6 | 444.2514 | 0.431391 | -1.21293 | 0.030607 | 1.42819 |
| Com_13539_pos | 13(2)-carboxypyropheophorbide a | C34 H34 N4 O5 | 578.2538 | 0.420888 | -1.24849 | 0.030644 | 1.572384 |
| Com_13441_pos | 3-Oxalomalic acid | C6 H6 O8 | 206.0067 | 2.267064 | 1.180825 | 0.030645 | 1.323704 |
| Com_5618_pos | Latanoprostene bunod | C27 H41 N O8 | 507.2828 | 0.115645 | -3.11223 | 0.030763 | 2.401486 |
| Com_24206_pos | ertapenem | C22 H25 N3 O7 S | 475.1408 | 0.465592 | -1.10286 | 0.03086 | 1.277919 |
| Com_1735_neg | Metildigoxin | C42 H66 O14 | 794.4477 | 4.465067 | 2.158682 | 0.03108 | 1.927643 |
| Com_1176_pos | Perflubron | C8 Br F17 | 497.8932 | 0.332491 | -1.58861 | 0.03109 | 1.533539 |
| Com_3243_neg | 2,3,14,20-Tetrahydroxy-22,23-epoxyergost-7-en-6-one | C28 H44 O6 | 476.3139 | 0.37884 | -1.40034 | 0.031209 | 1.504648 |
| Com_11487_neg | Tirofiban | C22 H36 N2 O5 S | 440.2346 | 0.525998 | -0.92687 | 0.031219 | 1.003678 |
| Com_10961_neg | Brivanib | C19 H19 F N4 O3 | 370.1453 | 0.469724 | -1.09011 | 0.031252 | 1.048305 |
| Com_6355_pos | (3Z,6E,8E,12Z,15Z,18Z)-3,6,8,12,15,18-Docosahexaen-10-ol | C22 H34 O | 314.2602 | 0.308204 | -1.69804 | 0.031336 | 2.34292 |
| Com_348_neg | VS1150000 | C18 H34 O4 | 314.2459 | 2.839592 | 1.505684 | 0.031707 | 1.79496 |
| Com_7438_neg | chelirubine | C21 H16 N O5 | 362.1037 | 0.337965 | -1.56505 | 0.031893 | 1.590016 |
| Com_3427_pos | Disinomenine | C38 H44 N2 O8 | 656.3115 | 8.28133 | 3.049862 | 0.031907 | 2.348409 |
| Com_10347_pos | Bevenopran | C20 H26 N4 O4 | 386.1957 | 0.288004 | -1.79584 | 0.032028 | 1.934878 |
| Com_9352_neg | (+/-)-Muscone | C16 H30 O | 238.2299 | 4.671701 | 2.223948 | 0.03218 | 2.04833 |
| Com_665_pos | (2E)-hexadecenoylcarnitine | C23 H43 N O4 | 397.3183 | 0.210626 | -2.24725 | 0.032425 | 1.993671 |
| Com_12100_pos | 3-[(3-Hydroxyheptanoyl)oxy]-4-(trimethylammonio)butanoate | C14 H27 N O5 | 289.1883 | 2.043883 | 1.031313 | 0.033102 | 1.05817 |
| Com_10021_pos | yatein | C22 H24 O7 | 400.1514 | 2.570872 | 1.362258 | 0.033113 | 1.546993 |
| Com_28379_pos | coenzyme Q2 | C19 H26 O4 | 318.1825 | 0.381032 | -1.39202 | 0.03318 | 1.874716 |
| Com_1282_neg | Promegestone | C22 H30 O2 | 326.2247 | 0.492169 | -1.02277 | 0.033238 | 1.238738 |
| Com_7190_neg | Carboprost | C21 H36 O5 | 368.2564 | 0.295259 | -1.75995 | 0.03341 | 1.458134 |
| Com_2158_pos | 240I539PWQ | C5 H14 N O6 P | 215.0555 | 3.582348 | 1.840905 | 0.03346 | 1.947697 |
| Com_6455_pos | TG(18:2(9Z,12Z)/18:2(9Z,12Z)/20:0)[iso3] | C59 H106 O6 | 910.7954 | 0.283753 | -1.81729 | 0.033717 | 1.857357 |
| Com_2551_pos | leminoprazole | C19 H23 N3 O S | 341.1576 | 0.442692 | -1.17562 | 0.034008 | 1.406638 |
| Com_14450_neg | Trimethylsilyl heptadecanoate | C20 H42 O2 Si | 342.2948 | 0.344607 | -1.53697 | 0.0341 | 1.527742 |
| Com_5417_neg | (2R)-2-Hydroxy-3-(phosphonooxy)propyl (13Z,16Z)-13,16-docosadienoate | C25 H47 O7 P | 490.3076 | 0.265967 | -1.91068 | 0.034358 | 1.671797 |
| Com_30389_pos | Norbuprenorphine | C25 H35 N O4 | 413.2548 | 2.366655 | 1.24285 | 0.034601 | 1.530437 |
| Com_12988_pos | ilomastat | C20 H28 N4 O4 | 388.2114 | 0.379735 | -1.39693 | 0.035383 | 1.412485 |
| Com_1074_neg | (2-Hydroxy-2-oxido-1,3,2-dioxaphospholan-4-yl)methyl palmitate | C19 H37 O6 P | 392.2328 | 0.319991 | -1.6439 | 0.035408 | 2.05988 |
| Com_11190_pos | Apigetrin | C21 H20 O10 | 432.1052 | 7.183504 | 2.844688 | 0.035611 | 2.019137 |
| Com_1887_neg | N(6)-Methyladenosine | C11 H15 N5 O4 | 281.1111 | 0.392285 | -1.35003 | 0.035685 | 1.277576 |
| Com_2114_pos | Tetranor-12(S)-HETE | C16 H26 O3 | 266.1878 | 2.718429 | 1.442773 | 0.036249 | 1.293844 |
| Com_22413_pos | ubiquinol | C49 H78 O4 | 730.5881 | 0.247427 | -2.01493 | 0.036483 | 1.598595 |
| Com_24573_pos | 3'-dehydro-ATP | C10 H14 N5 O13 P3 | 504.9804 | 0.451541 | -1.14707 | 0.036987 | 1.147111 |
| Com_2573_neg | Trifluoroacetic acid | C2 H F3 O2 | 113.9931 | 0.440213 | -1.18373 | 0.037067 | 1.101009 |
| Com_10216_pos | Glaucarubin | C25 H36 O10 | 496.2317 | 3.766627 | 1.913273 | 0.037436 | 1.824778 |
| Com_13381_pos | Acetyl hexamethyl tetralin | C18 H26 O | 258.1977 | 0.53042 | -0.91479 | 0.037472 | 1.118334 |
| Com_5120_neg | Azafrin | C27 H38 O4 | 426.2771 | 2.507993 | 1.326534 | 0.037552 | 1.380996 |
| Com_6607_neg | 7-deoxyloganin | C17 H26 O9 | 374.1581 | 5.061048 | 2.339436 | 0.037574 | 1.876576 |
| Com_7512_neg | tetrathionic acid | H2 O6 S4 | 225.8736 | 0.465057 | -1.10452 | 0.037687 | 1.464471 |
| Com_2105_neg | Hostmaniane | C13 H18 O5 | 254.1156 | 4.107719 | 2.038337 | 0.037811 | 2.375138 |
| Com_1006_pos | N-{3-[(4-Acetamidobutyl)amino]propyl}acetamide | C11 H23 N3 O2 | 229.1785 | 0.452058 | -1.14542 | 0.03802 | 1.24045 |
| Com_564_neg | Glutaric acid | C5 H8 O4 | 132.0424 | 2.50411 | 1.324298 | 0.038049 | 1.119085 |
| Com_2759_pos | lipoic acid | C8 H14 O2 S2 | 206.0431 | 3.754263 | 1.90853 | 0.038227 | 1.868059 |
| Com_6696_pos | Methymycin | C25 H43 N O7 | 469.3034 | 0.318905 | -1.6488 | 0.038377 | 1.564642 |
| Com_11282_pos | 5-Formiminotetrahydrofolic acid | C20 H24 N8 O6 | 472.1799 | 2.910504 | 1.541269 | 0.038581 | 1.440642 |
| Com_7647_pos | 7-Mercaptoheptanoylthreonine | C11 H21 N O4 S | 263.1187 | 0.198204 | -2.33494 | 0.03859 | 2.061663 |
| Com_10266_pos | Menogaril | C28 H31 N O10 | 541.195 | 6.708424 | 2.745974 | 0.038795 | 2.180918 |
| Com_4968_pos | (10S)-Juvenile hormone III diol | C16 H28 O4 | 284.1984 | 2.170607 | 1.118098 | 0.038915 | 1.041301 |
| Com_5126_neg | 3,4-Dimethylbenzoic acid | C9 H10 O2 | 150.0682 | 2.71196 | 1.439336 | 0.039147 | 1.248764 |
| Com_3393_pos | pro-gln | C10 H17 N3 O4 | 243.1211 | 1.970092 | 0.978263 | 0.039489 | 1.179115 |
| Com_607_pos | Batilol | C21 H44 O3 | 344.3282 | 0.109099 | -3.1963 | 0.039518 | 3.42133 |
| Com_1237_neg | 15S-hydroxyeicosatrienoic acid | C20 H34 O3 | 322.251 | 0.337517 | -1.56697 | 0.039537 | 1.437877 |
| Com_404_neg | Sarcostin | C21 H34 O6 | 382.2358 | 0.342601 | -1.5454 | 0.039559 | 1.830856 |
| Com_11199_neg | cortisol 21-sulfate | C21 H30 O8 S | 442.1663 | 0.264053 | -1.9211 | 0.039957 | 1.818298 |
| Com_339_neg | 4-(2-Aminophenyl)-2,4-dioxobutanoic acid | C10 H9 N O4 | 207.0534 | 3.64774 | 1.867003 | 0.039973 | 1.598307 |
| Com_23476_pos | (4S)-4-[(11-Carboxyundecanoyl)oxy]-4-(trimethylammonio)butanoate | C19 H35 N O6 | 373.2459 | 2.758772 | 1.464026 | 0.040231 | 1.234853 |
| Com_768_neg | soyasapogenol B 3-O-beta-glucuronide | C36 H58 O9 | 634.4094 | 4.359003 | 2.123998 | 0.040276 | 1.717173 |
| Com_13266_pos | Bendazac | C16 H14 N2 O3 | 282.0999 | 0.277834 | -1.8477 | 0.040515 | 1.420145 |
| Com_20466_pos | Crocetin | C20 H24 O4 | 328.1662 | 0.503899 | -0.98879 | 0.040564 | 1.036403 |
| Com_12911_pos | MFCD24849356 | C17 H17 N O4 | 299.1151 | 0.203101 | -2.29973 | 0.040598 | 1.674939 |
| Com_1484_neg | SB-206553 | C17 H16 N4 O | 292.1319 | 0.207207 | -2.27085 | 0.0407 | 1.642341 |
| Com_19939_pos | Ankorine | C19 H29 N O4 | 335.2089 | 2.369798 | 1.244764 | 0.040821 | 1.0805 |
| Com_606_pos | Acetyl-L-carnitine | C9 H17 N O4 | 203.1155 | 2.090859 | 1.064096 | 0.040836 | 1.473283 |
| Com_7118_pos | Haloperidol decanoate | C31 H41 Cl F N O3 | 529.2746 | 2.397168 | 1.261331 | 0.040986 | 1.855803 |
| Com_848_pos | N-LACTOYL ETHANOLAMINE PHOSPHATE | C5 H12 N O6 P | 213.0401 | 4.190789 | 2.067222 | 0.04117 | 2.311988 |
| Com_17920_pos | 2,3,4,9-Tetrahydro-1H-beta-carboline-1,3-dicarboxylic acid | C13 H12 N2 O4 | 260.0791 | 2.591198 | 1.373619 | 0.041459 | 1.149398 |
| Com_10915_neg | nifurquinazol | C16 H16 N4 O5 | 344.1119 | 0.333 | -1.5864 | 0.04172 | 1.388374 |
| Com_371_pos | Creatinine | C4 H7 N3 O | 113.059 | 2.996191 | 1.58313 | 0.041755 | 1.52664 |
| Com_318_pos | L-Prolinamide | C5 H10 N2 O | 114.0793 | 2.145579 | 1.101367 | 0.041857 | 1.04382 |
| Com_5670_pos | 2-methoxy-5-methyl-3-(2-methylbut-3-en-2-yl)chromen-4-one | C16 H18 O3 | 296.0823 | 0.029991 | -5.05931 | 0.041938 | 3.315936 |
| Com_7816_neg | 2-(2-Acetoxy-2-oxoethyl)-2-hydroxysuccinic acid | C8 H10 O8 | 234.0375 | 0.391326 | -1.35356 | 0.042011 | 1.366415 |
| Com_1575_neg | 7-Methylxanthine | C6 H6 N4 O2 | 166.0479 | 3.690993 | 1.884009 | 0.042015 | 1.523736 |
| Com_8115_pos | Diphenylmercury | C12 H10 Hg | 356.0493 | 0.402783 | -1.31192 | 0.042046 | 1.1431 |
| Com_21856_pos | Pibutidine | C19 H24 N4 O3 | 356.1852 | 0.426742 | -1.22856 | 0.042207 | 1.113645 |
| Com_7176_pos | trans-Cinnamaldehyde | C9 H8 O | 132.0575 | 3.153119 | 1.656779 | 0.042249 | 1.293629 |
| Com_18839_pos | Heneicosylic acid | C21 H42 O2 | 326.3177 | 0.285179 | -1.81006 | 0.042738 | 1.549194 |
| Com_16226_pos | 2,2'-Dihydroxy-4,4'-diphenyl-3H,3'H-1,1'-biphenalene-3,3'-dione | C38 H22 O4 | 542.1534 | 5.841242 | 2.546275 | 0.042796 | 1.873065 |
| Com_232_neg | Genistein | C15 H10 O5 | 270.0529 | 2.738566 | 1.453421 | 0.04283 | 1.961879 |
| Com_2084_pos | decanoylcarnitine | C17 H33 N O4 | 315.2402 | 0.387459 | -1.36788 | 0.042861 | 1.494678 |
| Com_2531_pos | pyricarbate | C11 H15 N3 O4 | 253.1054 | 2.805541 | 1.488279 | 0.04302 | 1.264974 |
| Com_1101_pos | N-lauroylglycine | C14 H27 N O3 | 257.1986 | 2.288066 | 1.194129 | 0.043195 | 1.446444 |
| Com_1189_neg | prostaglandin H2 2-glyceryl ester | C23 H38 O7 | 426.2618 | 0.131561 | -2.9262 | 0.043596 | 1.842508 |
| Com_1898_pos | L-2-succinylamino-6-oxoheptanedioic acid | C11 H15 N O8 | 289.079 | 0.499285 | -1.00207 | 0.043612 | 1.308482 |
| Com_2385_neg | Lenvatinib | C21 H19 Cl N4 O4 | 426.11 | 2.190576 | 1.13131 | 0.043673 | 1.07617 |
| Com_562_neg | Phloionolic acid | C18 H36 O5 | 332.2565 | 7.401213 | 2.887762 | 0.043741 | 2.176704 |
| Com_5401_pos | 2-Acetamido-2-deoxy-D-glucono-1,5-lactone | C8 H13 N O6 | 219.0738 | 2.412074 | 1.270274 | 0.043967 | 1.135911 |
| Com_14887_pos | jamaicamide A | C27 H36 Br Cl N2 O4 | 566.1526 | 3.352814 | 1.745372 | 0.044169 | 1.531323 |
| Com_10683_pos | fluphenazine depot | C32 H44 F3 N3 O2 S | 591.3124 | 0.427333 | -1.22657 | 0.044207 | 1.307504 |
| Com_32_neg | Deoxycholic acid | C24 H40 O4 | 392.293 | 2.982828 | 1.576681 | 0.04438 | 1.463548 |
| Com_433_pos | Bilirubin | C33 H36 N4 O6 | 584.2622 | 0.156546 | -2.67534 | 0.044696 | 2.525237 |
| Com_22813_pos | (3E)-4,8-Dimethyl-1,3,7-nonatriene | C11 H18 | 150.1409 | 0.37861 | -1.40122 | 0.044756 | 1.434407 |
| Com_9291_pos | 3-[3,5-Dihydroxy-4-(sulfooxy)phenyl]-2-oxopropanoic acid | C9 H8 O9 S | 291.99 | 1.773442 | 0.826552 | 0.04502 | 1.005721 |
| Com_3349_neg | terameprocol | C22 H30 O4 | 358.2146 | 0.402898 | -1.31151 | 0.045067 | 1.459007 |
| Com_1046_neg | Glycoursodeoxycholic acid | C26 H43 N O5 | 449.3144 | 2.577985 | 1.366244 | 0.045109 | 1.501799 |
| Com_12047_neg | Withaferine | C28 H38 O6 | 470.2652 | 1.842477 | 0.881646 | 0.045453 | 1.113921 |
| Com_2092_pos | Jasmonic acid | C12 H18 O3 | 210.1254 | 2.659925 | 1.411385 | 0.045475 | 1.538234 |
| Com_44_pos | LysoPC(P-18:0) | C26 H54 N O6 P | 507.3683 | 0.519635 | -0.94443 | 0.045597 | 1.257221 |
| Com_5265_pos | Biotin l-Sulfoxide | C10 H16 N2 O4 S | 260.0825 | 0.46434 | -1.10675 | 0.045648 | 1.1437 |
| Com_8121_pos | 1-Eicosapentaenoyl-2-docosahexaenoyl-sn-glycero-3-phosphoethanolamine | C47 H72 N O8 P | 809.4988 | 3.268427 | 1.708597 | 0.045738 | 1.554607 |
| Com_6855_pos | 17,21-Dihydroxypregnenolone | C21 H32 O4 | 348.2291 | 0.255008 | -1.97138 | 0.045865 | 1.351209 |
| Com_20568_pos | sempervirene | C19 H16 N2 | 272.132 | 2.52976 | 1.339 | 0.045892 | 1.223441 |
| Com_1420_neg | 10,16-Dihydroxyhexadecanoic acid | C16 H32 O4 | 288.23 | 2.076018 | 1.053819 | 0.045899 | 1.208686 |
| Com_11023_neg | Retosiban | C27 H34 N4 O5 | 494.2514 | 0.249675 | -2.00188 | 0.045989 | 1.409627 |
| Com_5037_pos | 3-[(12-Hydroxyoctadecanoyl)oxy]-4-(trimethylammonio)butanoate | C25 H49 N O5 | 443.3604 | 0.302259 | -1.72614 | 0.046013 | 1.43477 |
| Com_16499_pos | 4-Hydroxyprolylmethionine | C10 H18 N2 O4 S | 262.0978 | 2.659534 | 1.411173 | 0.046099 | 1.233069 |
| Com_2041_pos | N-hexadecanoylsphinganine | C34 H69 N O3 | 539.5273 | 0.222871 | -2.16572 | 0.04616 | 2.580153 |
| Com_8244_pos | Itarnafloxin | C35 H33 F N6 O3 | 604.2601 | 6.305482 | 2.656607 | 0.046344 | 1.943844 |
| Com_11424_pos | MFCD05664273 | C8 H6 O S | 150.0138 | 0.199522 | -2.32538 | 0.046359 | 1.691832 |
| Com_9520_pos | Jadomycin B | C30 H31 N O9 | 549.1985 | 0.318113 | -1.65239 | 0.047405 | 1.902208 |
| Com_8992_pos | Alacepril | C20 H26 N2 O5 S | 406.1579 | 0.550968 | -0.85996 | 0.047692 | 1.136893 |
| Com_2440_pos | Lotaustralin | C11 H19 N O6 | 261.1206 | 2.225712 | 1.154267 | 0.047711 | 1.006031 |
| Com_2146_neg | 2-Phenylethyl 6-O-beta-D-xylopyranosyl-beta-D-glucopyranoside | C19 H28 O10 | 416.1685 | 3.974367 | 1.990725 | 0.047807 | 2.345768 |
| Com_12043_neg | Akeboside Ste | C47 H76 O16 | 896.5169 | 2.000785 | 1.000566 | 0.048114 | 1.005652 |
| Com_271_neg | 2-Acetamido-2-deoxyglucose | C8 H15 N O6 | 221.09 | 0.419566 | -1.25303 | 0.048289 | 1.364974 |
| Com_10662_pos | shanzhiside | C16 H24 O11 | 392.1325 | 0.355426 | -1.49238 | 0.048947 | 1.272795 |
| Com_3530_pos | Ranelic acid | C12 H10 N2 O8 S | 342.0173 | 3.123886 | 1.643342 | 0.049168 | 1.571217 |
| Com_736_neg | pateamine | C31 H45 N3 O4 S | 555.314 | 0.551449 | -0.8587 | 0.049287 | 1.101169 |
| Com_804_neg | alpha-Methyl D-mannoside | C7 H14 O6 | 194.0791 | 2.709488 | 1.43802 | 0.049756 | 1.688565 |

Note:

Abbreciations: FC, Fold Change; VIP, Variable Importance in the Projection
